# Carbonic anhydrase inhibitors in patients with respiratory failure and metabolic alkalosis: a systematic review and meta-analysis of randomized controlled trials

**DOI:** 10.1186/s13054-018-2207-6

**Published:** 2018-10-29

**Authors:** Bassem Y Tanios, Maryam O Omran, Carlos Noujeim, Tamara Lotfi, Samir S Mallat, Pierre K Bou-Khalil, Elie A Akl, Houssam S Itani

**Affiliations:** 1 Division of Nephrology and Hypertension, American University of Beirut Medical Center, Beirut, Lebanon; 2 Faculty of Health Sciences, American University of Beirut, Beirut, Lebanon; 3 Division of pulmonary and critical care, Keserwan Medical Center, Ghazir, Lebanon; 4 Clinical Research Institute, and Faculty of Health Sciences, American University of Beirut, Beirut, Lebanon; 5 Division of Pulmonary and Critical Care, American University of Beirut Medical Center, Beirut, Lebanon; 6 Department of Internal Medicine, American University of Beirut, Beirut, Lebanon; 7 Division of Nephrology and Hypertension, Makassed General Hospital, Beirut, Lebanon; 8 Department of Internal Medicine, American University of Beirut Medical Center, PO Box 11-0236, Riad El-Solh, Beirut, 1107 2020 Lebanon

**Keywords:** Carbonic anhydrase inhibitors, Respiratory failure, Metabolic alkalosis, Mechanical ventilation, Systematic review

## Abstract

**Background:**

Metabolic alkalosis is common in patients with respiratory failure and may delay weaning in mechanically ventilated patients. Carbonic anhydrase inhibitors block renal bicarbonate reabsorption, and thus reverse metabolic alkalosis. The objective of this systematic review is to assess the benefits and harms of carbonic anhydrase inhibitor therapy in patients with respiratory failure and metabolic alkalosis.

**Methods:**

We searched the following electronic sources from inception to August 2017: the Cochrane Central Register of Controlled Trials (CENTRAL), MEDLINE, EMBASE, and SCOPUS. Randomized clinical trials were included if they assessed at least one of the following outcomes: mortality, duration of hospital stay, duration of mechanical ventilation, adverse events, and blood gas parameters. Teams of two review authors worked in an independent and duplicate manner to select eligible trials, extract data, and assess risk of bias of the included trials. We used meta-analysis to synthesize statistical data and then assessed the certainty of evidence using the GRADE methodology.

**Results:**

Six eligible studies were identified with a total of 564 participants. The synthesized data did not exclude a reduction or an increase in mortality (risk ratio (RR) 0.94, 95% confidence interval (CI) 0.57 to 1.56) or in duration of hospital stay (mean difference (MD) 0.42 days, 95% CI −4.82 to 5.66) with the use of carbonic anhydrase inhibitors. Carbonic anhydrase inhibitor therapy resulted in a decrease in the duration of mechanical ventilation of 27 h (95% CI −50 to −4). Also, it resulted in an increase in PaO_2_ (MD 11.37 mmHg, 95% CI 4.18 to 18.56) and a decrease in PaCO_2_ (MD −4.98 mmHg, 95% CI −9.66, −0.3), serum bicarbonate (MD −5.03 meq/L, 95% CI −6.52 to −3.54), and pH (MD −0.04, 95% CI −0.07 to −0.01). There was an increased risk of adverse events in the carbonic anhydrase inhibitor group (RR 1.71, 95% CI 0.98 to 2.99). Certainty of evidence was judged to be low for most outcomes.

**Conclusion:**

In patients with respiratory failure and metabolic alkalosis, carbonic anhydrase inhibitor therapy may have favorable effects on blood gas parameters. In mechanically ventilated patients, carbonic anhydrase inhibitor therapy may decrease the duration of mechanical ventilation. A major limitation of this finding was that only two trials assessed this clinically important outcome.

**Electronic supplementary material:**

The online version of this article (10.1186/s13054-018-2207-6) contains supplementary material, which is available to authorized users.

## Background

Metabolic alkalosis is common in patients with respiratory failure [1, 2]. There is evidence that increased pH level in the cerebrospinal fluid may depress respiratory drive and may delay weaning in patients with respiratory failure on mechanical ventilation [3, 4].

Carbonic anhydrase inhibitors (CAI) (such as acetazolamide, methazolamide, and dichlorphenamide) block renal bicarbonate reabsorption, and thus reverse metabolic alkalosis [3]. However, uncertainty remains about their effects in the setting of respiratory failure with concurrent metabolic alkalosis on duration of hospitalization, mechanical ventilation (MV), or noninvasive positive-pressure ventilation (NIPPV), and mortality [3].

A Cochrane systematic review on the use of CAI for hypercapnic ventilatory failure in chronic obstructive pulmonary disease (COPD) identified four eligible trials. The review found that acetazolamide therapy resulted in a significant improvement in PO_2_ and a nonsignificant decrease in PaCO_2_. However, the included studies had a limited number of participants (84 patients), had short-term follow-ups, did not assess clinically important outcomes such as duration of hospitalization and mortality, and none included patients on NIPPV or MV [5].

Since the publication of the Cochrane review in 2001, several trials have been published [6–8]. In a multicenter randomized controlled trial (RCT), acetazolamide therapy in 382 patients with COPD and metabolic alkalosis on mechanical ventilation resulted in a 16-h decrease in the duration of mechanical ventilation compared with placebo (95% confidence interval (CI) −36.5 to 4.0 h; *p* = 0.17) [6]. A smaller trial with 22 patients with COPD with respiratory failure, metabolic alkalosis, and on NIPPV, found that acetazolamide therapy significantly reduced the duration of NIPPV compared with a matched control group (6 ± 8 versus 19 ± 19 days; *p* = 0.03) [7].

Given the current uncertainty about the benefits and harms of using CAI in patients with respiratory failure and metabolic alkalosis, it would be informative for clinical practice to synthesize and critically appraise the current body of evidence.

### Objective

The objective of this systematic review was to assess the benefits and harms of carbonic CAI therapy in patients with respiratory failure and metabolic alkalosis.

## Methods

The detailed methods are included in Additional file 1.

### Eligibility criteria

RCTs were included if they recruited patients with respiratory failure and concurrent metabolic alkalosis (as defined by the individual trial), including patients on MV or NIPPV. In addition, the trial should have compared CAI to either placebo or usual care. All co-interventions should have been similar for the two comparison groups.

The primary outcomes of interest were duration of hospital stay, duration of MV or NIPPV, mortality, and adverse events. Secondary outcomes included the blood gases parameters PaCO_2_, PO_2_, HCO_3_, and PH.

### Search strategy

**Fig. 1:**
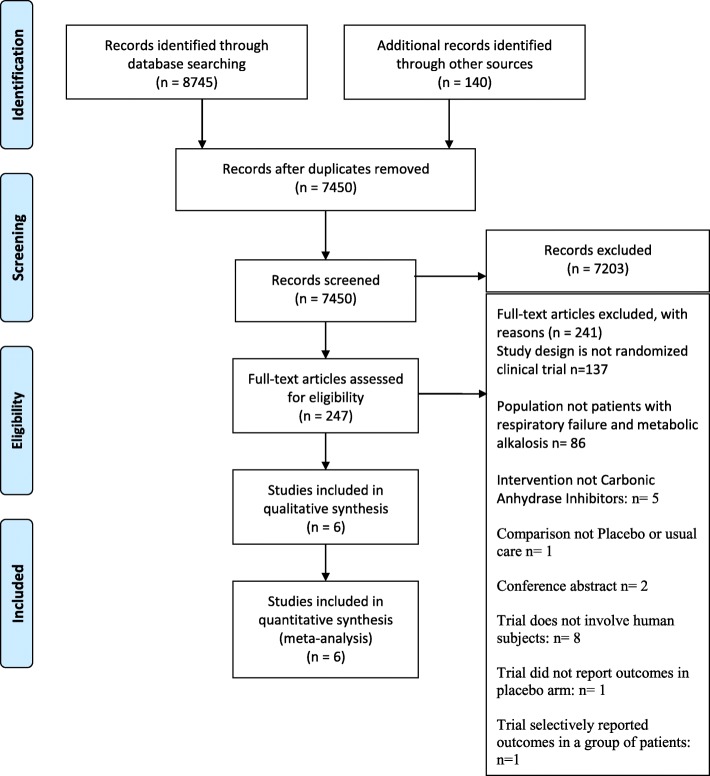
PRISMA study flowchart

**Fig. 2:**
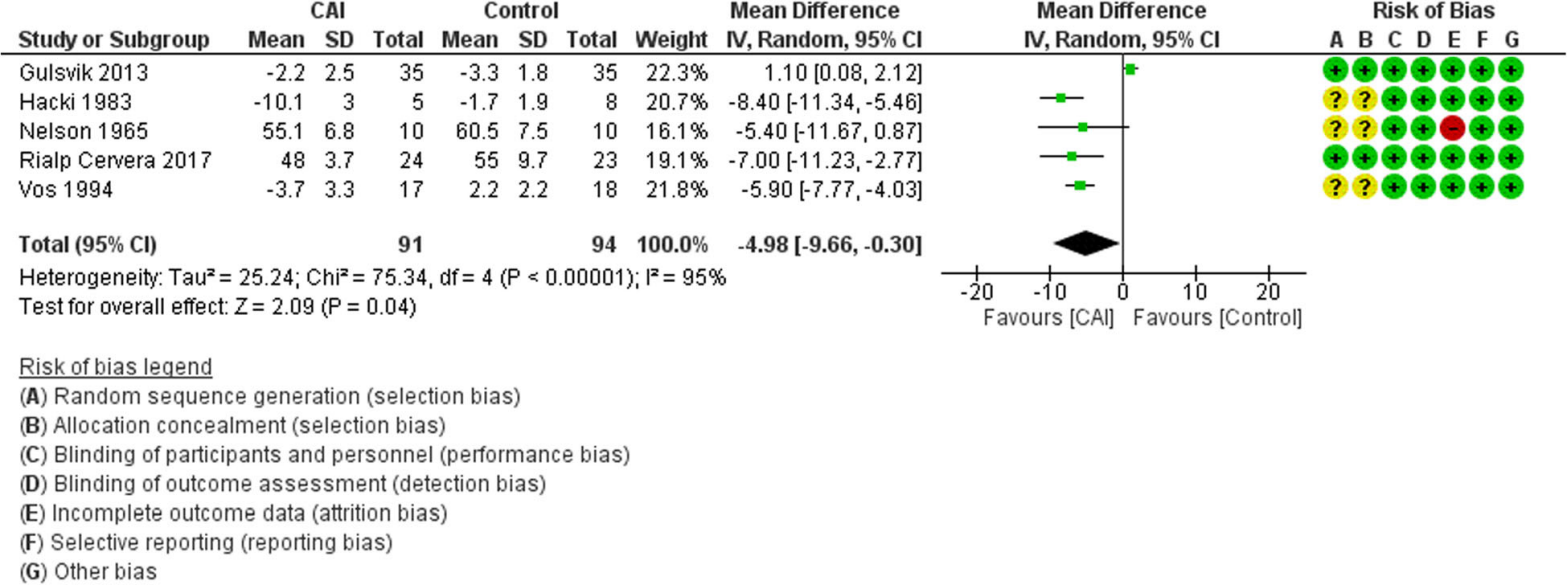
Forest plot for the effect of carbonic anhydrase inhibitors (CAI) vs control on PaCO_2_. CI confidence interval, IV Inverse variance, SD standard deviation

**Fig. 3:**
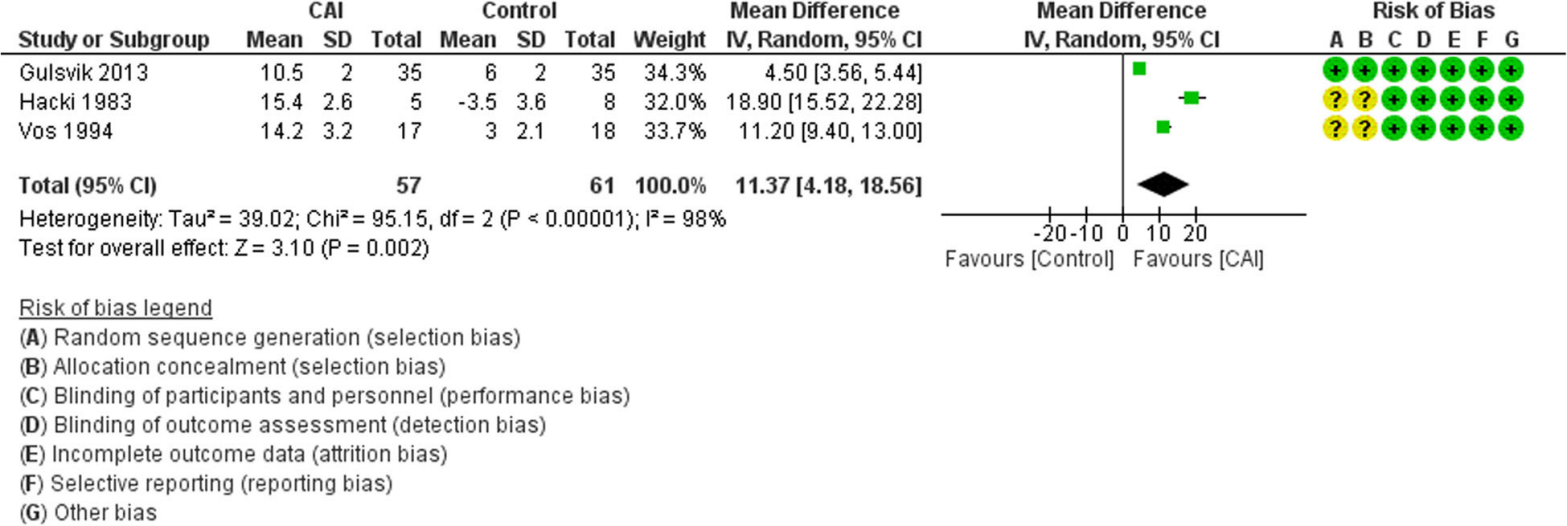
Forest plot for the effect of carbonic anhydrase inhibitors (CAI) vs control on PaO_2_. CI confidence interval, IV Inverse variance, SD standard deviation

**Fig. 4:**
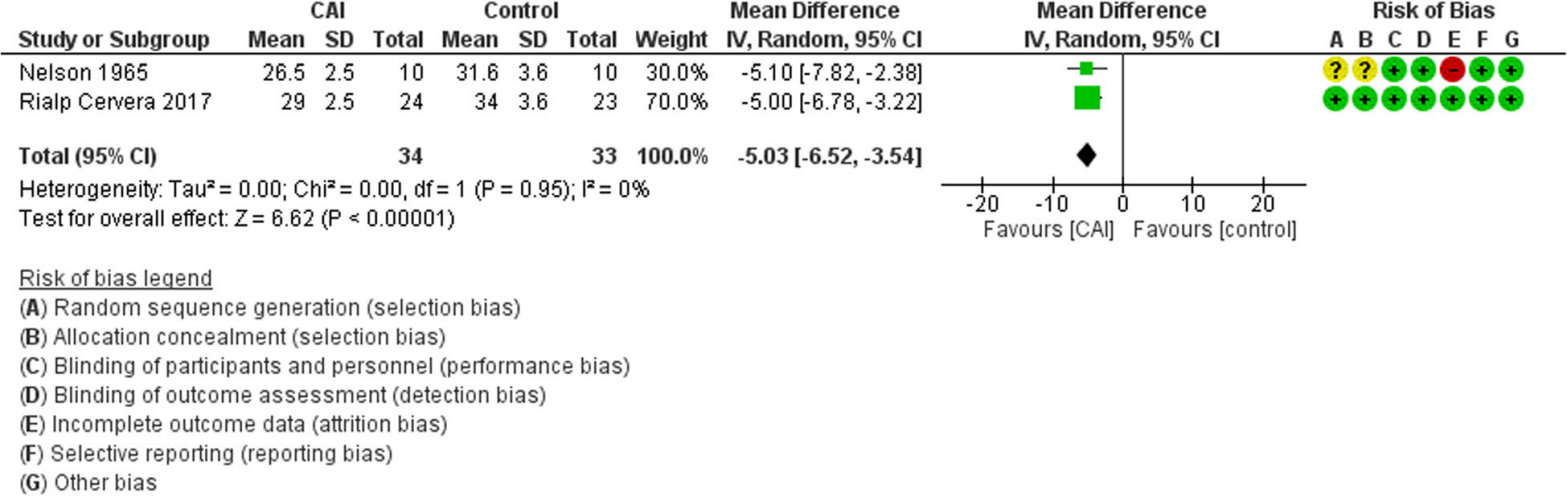
Forest plot for the effect of carbonic anhydrase inhibitors (CAI) vs control on serum bicarbonate. CI confidence interval, IV Inverse variance, SD standard deviation

We searched the following electronic databases from inception to August 2017: the Cochrane Central Register of Controlled Trials (CENTRAL), MEDLINE, EMBASE, and SCOPUS. Figures 1 to 4 in Additional file 2 detail the electronic search strategy. There were no language or date restrictions. We also screened the reference lists of included trials and identified related systematic reviews. The search strategy did not include attempts at collecting unpublished data.

### Selection process

Teams of two review authors (BYT and CN, and HSI and MOO) screened independently and in duplicate the abstract and title of every record captured by the searches. We retrieved the full texts for all citations judged as potentially eligible by at least one reviewer.

The teams of two reviewers then assessed the full texts for eligibility using a standardized screening form.

A PRISMA (preferred reporting items for systematic reviews and meta-analyses) flow-chart was used to summarize the results of the selection process [9] (Fig. 1).

### Data extraction

We extracted information about the study design, the clinical characteristics of the trial (population, intervention, comparator, and outcomes), funding, and conflicts of interest of the authors.

### Assessment of risk of bias

Risk of bias was assessed using The Cochrane Collaboration’s Risk of Bias tool [10, 11]. The following criteria were used: random sequence generation (selection bias), allocation concealment (selection bias), blinding of participants, providers, data collectors, outcome adjudicators, and data analysts (performance bias and detection bias), incomplete outcome data (attrition bias), selective outcome reporting (reporting bias), and other biases (including early stopping for benefit).

Risk of bias criteria was judged as ‘low risk’, ‘high risk’ or ‘unclear risk’ as described in the *Cochrane Handbook for Systematic Reviews of Interventions* [10].

### Data analysis

For dichotomous data, we used the risk ratio (RR) with 95% CIs. For continuous outcomes data, we used, whenever possible, the mean change score from baseline to follow-up for each intervention group.

One of the of the included trials (Nelson and Wallace, 1965 [12]) did not report standard deviations (SDs) in the assessment of the outcomes for PaCO_2_ and serum bicarbonate. Therefore, we used the median SD from the other included trials that reported SDs for these outcomes, as described in Furukawa et al. [13]. In another trial (Faisy et al., 2016 [14]), the authors did not report means and SDs and so these were extrapolated respectively from the reported medians (mean = median) and interquartile range (IQR) (SD = IQR/1.35) [10]. In Hacki et al., 1983 [15], outcomes data were extracted from a graph in the report using the WebPlotDigitizer tool [16].

We pooled data using the random-effects model for the primary meta-analyses [17]. Heterogeneity (inconsistency) between study results was assessed using the *I*^2^ statistic. An *I*^2^ value of 50% or more was indicative of a considerable level of heterogeneity [10]. To explain any heterogeneity, we planned to conduct subgroup analyses based on the following variables: specific type and dose of CAI, etiology of respiratory failure, spontaneously breathing patients or on MV or NIPPV, and severity of metabolic alkalosis.

We also planned to perform sensitivity analyses to explore the influence of the following factors (when applicable) on pooled effect sizes: restricting the analyses to studies with low risk of bias, restricting the analyses to studies with longer follow-up, and assessing the impact of missing data. [18–21].

### Assessment of certainty of the evidence

Certainty of the evidence for each outcome was assessed using the Grading of Recommendations Assessment, Development and Evaluation (GRADE) approach [22]. This approach classifies the certainty of evidence into four categories: high, moderate, low, and very low. It takes into account the following factors: risk of bias [23], imprecision, inconsistency [24], indirectness [25], and publication bias [26]. We developed a Summary of Findings (SoF) table using the GRADEpro/GDT tool [27].

## Results

### Search results

Figure 1 shows the study flow chart. Out of 7450 screened citations, six eligible studies were identified with a total of 578 participants.

### Included studies

Table 1 summarizes the characteristics of the included studies. Five trials were in the English language, and one trial was in German. All studies were randomized. Four trials used a parallel group design [6, 8, 28, 29], one trial used a cross-over design [12], and one trial [15] used a cross-over design during the short-term intervention and a parallel design during long-term intervention.

**Table 1:** Characteristics of included studies

Study name	Study design	Participants	Intervention	Control	Outcomes assessed
Faisy et al., 2016 [6]	Randomized double blind multicenter trial	*N* = 382 Mean age = 69 Females = 29% (*N* = 110) Country, France Mechanically ventilated patients mostly secondary to community-acquired pneumonia 43% (*N* = 166), bronchitis 18% (*N* = 70), and left ventricular insufficiency 19% (*N* = 75), and metabolic alkalosis (serum HCO_3_ > 26 meq/l and arterial PH ≥ 7.35 mmHg)	Acetazolamide 500 mg or 1000 mg (when loop diuretics were co-prescribed) intravenously twice per day for 28 days	10 ml normal saline twice daily for 28 days	Duration of invasive ventilation Changes in serum HCO_3_ Arterial blood gases PFTs Weaning duration Ventilator-associated pneumonia episodes Use of noninvasive ventilation after extubation Successful weaning Duration of ICU stay ICU mortality Adverse events
Rialp Cervera et al., 2017 [8]	Multicenter, randomized, controlled, double-blind study	*N* = 47 Mean age 67 Females 23% (*N* = 11) Country, Spain COPD or OHS with invasive MV, and metabolic alkalosis (PH > 7.35 with plasma HCO_3_ > 28 mmol/l)	Capsules of 500 mg of acetazolamide by nasogastric tube for 28 days	Placebo one tablet once daily by nasogastric tube for 28 days	Duration of MV Duration of weaning Need for tracheostomy Application of postextubation noninvasive ventilation, Re-intubation in 48 h Duration of ICU stay Duration of hospital stay Hospital mortality Adverse effects Acid base balance
Nelson and Wallace, 1965 [12]	Double blind, controlled, cross-over design	*N* = 12 Mean age: 52 Female = 16% (*N* = 2) Country, Northern Ireland Outpatient COPD with either an arterial oxygen saturation of less than 90% or PCO_2_ of 53 mmHg or more and metabolic alkalosis with CO_2_ content 31.6 at baseline.	Dichlorphenamide 50 mg four times per day for 3 consecutive fortnights	Placebo 1 tablet four times per day for 3 consecutive fortnights	Oxygen saturation Blood gases parameters Symptomatic effects Adverse events
Hacki et al.,1983 [15]	Randomized, double blind, controlled trial, sequential design (cross-over then parallel group)	*N* = 14 Patients with COPD who met the following conditions: PO_2_ < 60 mmHg, PCO_2_ > 45 mmHg, and pH > 7.38	Acute term intervention (cross-over design): Acetazolamide 250 mg twice daily with cross-over between intervention and placebo at day 3,6,9, Long-term intervention (parallel group design): re-randomization at day 12 and treatment of one group with acetazolamide 250 mg twice daily for a median of 4.5 months	Placebo twice daily	Acute phase of trial: PaO_2_ and PCO_2_ levels by ABG on days 0, 3, 6, 9, and 12 Pulmonary function tests on days 0 and 12 Weight on days 0 and 12 Long-term phase: blood gases after 4.5 months follow-up
Vos et al., 1994 [29]	Randomized, double blind, placebo controlled	*N* = 53 Mean age: 65 Females = 26% (*N* = 14) Country, the Netherlands Outpatient COPD with PaO_2_ < 8.5 kPa and metabolic alkalosis with base excess: 6.6 mmol/l at baseline	Acetazolamide 250 mg twice per day for one week	Placebo tablets twice per day for one week	PaO_2_, pH, PaCO_2_, base excess, Hypercapnic ventilatory response Hypoxic ventilatory response Quality of sleep Beneficial effects according to patients Side effects
Gulsvik et al., 2013 [28]	Randomized, placebo-controlled, double-blind, parallel group trial	N = 70 Mean age: 73.5 Female: 63%, (*N* = 44) Country, Norway Hospitalized COPD patients with PaO_2_ ≤ 8 kPa and/or PaCO_2_ ≥ 7 kPa, and metabolic alkalosis with base excess ≥8 mmol/L 13 patients received noninvasive ventilation	Acetazolamide tablets 250 mg three times per day for 5 days	Placebo tablets three times per day for 5 days	Primary outcome: PaO_2_ Secondary outcomes: PaCO_2_, base excess, pH, total number of days in hospital, adverse effects

*ABG* arterial blood gases, *COPD* chronic obstructive pulmonary disease, *ICU* intensive care unit, *MV* mechanical ventilation, *OHS* obesity-hypoventilation syndrome, *PFT* pulmonary function test

Only two trials (Faisy et al. and Rialp Cervera et al.) included patients on mechanical ventilation [6, 8], while the other four trials included outpatients and inpatients not on mechanical ventilation. Four trials included COPD patients exclusively [12, 28–30]. In the study by Rialp Cervera et al., 90% of participants had COPD while 10% had obesity hypoventilation syndrome [8]. Faisy et al. included patients on mechanical ventilation secondary mostly to community-acquired pneumonia and bronchitis [6]. Five trials evaluated acetazolamide, while one trial (Nelson and Wallace) evaluated dichlorphenamide [12]. Additional file 2 (Table S5) details the funding and conflicts of interest of authors in the included trials.

### Risk of bias in included trials

Additional file 2 (Figure S1) and Table 2 summarize the risk of bias assessment in the included trials. Most of the included trials were judged to have a low risk of bias for most of the criteria. An unclear risk of bias was judged for some trials [6, 12, 29, 30], especially when methods of random sequence generation and allocation concealment were not specified. A high-risk attrition bias was judged for Nelson and Wallace because 12 patients were included in the final analysis out of the initial 19 patients included in the trial [12]. A high “other risk of bias” was also judged for Faisy et al. [6], taking into consideration that we extrapolated means and SDs as described above.

**Table 2:** Risk of bias in included studies

Study name	Random sequence generation	Allocation concealment	Blinding	Completeness of data	Selective outcome reporting	Other bias
Faisy et al., 2016 [6]	Low risk “The randomization sequence was programmed in advance and generated by a statistician independent of the study”	Low risk “Patients were randomized via a computer-generated assignment sequence in a centralized blinded fashion”	Low risk “Double-blind placebo-controlled trial” No details provided on which specific groups were blinded	Low risk “One patient from each group was excluded”	Low risk All outcomes listed in the methods section are reported in the results section	High risk Means and standard deviations were extrapolated from medians and interquartile ranges
Rialp Cervera et al., 2017 [8]	Low risk “Randomization was based on computer-generated random numbers”	Low risk “Treatment and placebo capsules were prepared, packaged and blinded in a centralized hospital pharmacy and distributed to all ICUs”	Low risk “Double-blind study” “Investigators, patients and caregivers were unaware of the randomization list”	Low risk “All enrolled patients completed the trial and were included in the final analysis”	Low risk All outcomes listed in the methods section are reported in the results section	Low risk
Nelson and Wallace, 1965 [12]	Unclear risk Method of random sequence generation not specified	Unclear risk Method of allocation concealment not specified	Low risk “Active and placebo tablets were identical in appearance, and their identity was unknown to the patients and to the assessors during the trial”	High risk “Nineteen patients began the trial, but only 12 were included in the final analysis”	Low risk All outcomes listed in the methods section are reported in the results section	Low risk
Haecki et al., 1983 [15]	Unclear risk Method of random sequence generation not specified	Unclear risk Method of allocation concealment not specified	Low risk “Double blind trial” No details provided on which specific groups were blinded	Low risk “One patient refused follow-up”	Low risk All outcomes listed in the methods section are reported in the results section	Low risk
Vos et al., 1994 [29]	Unclear risk Method of random sequence generation not specified	Unclear risk Method of allocation concealment not specified	Low risk of bias “Double blind trial” No details provided on which specific groups were blinded	Low risk “All patients were studied during three nights”	Low risk All outcomes listed in the methods section are reported in the results section	Low risk
Gulsvik et al., 2013 [28]	Low risk “Randomization was based on computer-generated random numbers”	Low risk “Patients were allocated randomly on a 1:1 basis to a sealed and numbered box containing either acetazolamide or placebo tablets with similar size and colour”	Low risk “placebo controlled and double-blind parallel group trial”	Low risk All enrolled patients were included in the final analysis	Low risk All outcomes listed in the methods section are reported in the results section	Low risk

*ICU* intensive care unit

### Effects of the intervention

#### Mortality

The analysis based on the two trials that reported mortality did not exclude a reduction or an increase in mortality (RR 0.94, 95% CI 0.57 to 1.56; *I*^2^ = 0%). Certainty of evidence was judged to be low due to very serious imprecision (Additional file 2: Figure S2; see the SoF in Table 3).

**Table 3:** Summary of findings

Outcomes	Anticipated absolute effects (95% CI)		Relative effect (95% CI)	No. of participants (studies)	Certainty of the evidence (GRADE)
	Risk with control	Risk with carbonic anhydrase			
Duration of hospital stay (days)		The mean duration of hospital stay in the intervention group was 0.42 days more (4.82 fewer to 5.66 more)	–	117 (2 RCTs)	⨁⨁◯◯ LOW^a^
Duration of mechanical ventilation (h)		The mean duration of mechanical ventilation in the intervention group was 27.09 h lower (50.11 lower to 4.08 lower)	–	427 (2 RCTs)	⨁⨁◯◯ LOW^b,c^
Mortality	130 per 1000	122 per 1000 (74 to 202)	RR 0.94 (0.57 to 1.56)	427 (2 RCTs)	⨁⨁◯◯ LOW^a^
Adverse events	78 per 1000	133 per 1000 (76 to 232)	RR 1.71 (0.98 to 2.99)	508 (5 RCTs)	⨁⨁⨁◯ MODERATE^a^

Carbonic anhydrase inhibitors were compared with control for respiratory failure with metabolic alkalosis

Patient or population: respiratory failure with metabolic alkalosis

Intervention: carbonic anhydrase inhibitors

Comparison: control

*CI* confidence interval, *RCT* randomized controlled trial, *RR* risk ratio

^a^ Wide confidence intervals, very serious imprecision

^b^ Wide confidence intervals, serious imprecision

^c^ We extrapolated mean and standard deviation from the median and interquartile ranges reported in the trial

#### Duration of hospital stay

Only two trials assessed the duration of hospital stay, and the pooled analysis did not exclude a reduction or an increase in duration of hospital stay (mean difference (MD) 0.42 days, 95% CI –4.82 to 5.66; *I*^2^ = 33%) (Additional file 2: Figure S3). Certainty of evidence was judged to be low due to very serious imprecision (Table 3).

#### Duration of MV or NIPPV

Pooled data from the two trials that included patients on MV found that CAI therapy resulted in a 27.09-h mean reduction in duration of MV (95% CI –50.11 to −4.08; *I*^2^ = 0%) (Additional file 2: Figure S4). Certainty of evidence was judged to be low due to imprecision and serious risk of bias (Table 3).

#### PaCO_2_

The main analysis included five trials and excluded Faisy et al. [6] because they reported daily mean change in PaCO_2_. The meta-analysis showed that CAI therapy resulted in a mean reduction of −4.98 mmHg in PaCO_2_ (95% CI −9.66 to −0.30; *I*^2^ = 95%) (Fig. 2). The result did not change substantially after including Faisy et al. using the standardized mean difference (SMD) (Additional file 2: Figure S5).

#### PaO_2_

The main analysis included three trials and excluded Faisy et al. and Rialp Cervera et al. who reported Pa/FiO_2_, and Nelson and Wallace who did not report on this outcome. The meta-analysis showed that CAI therapy resulted in a mean increase of 11.37 mmHg in PaO_2_ (95% CI 4.18 to 18.56; *I*^2^ = 98%) (Fig. 3). The results still favored the CAI group when we included Faisy et al. and Rialp Cervera et al. using SMD (Additional file 2: Figure S6).

#### Serum bicarbonate

The main analysis included two trials and showed a mean reduction of 5 meq/L in serum bicarbonate in the CAI group (95% CI −6.52 to −3.54; *I*^2^ = 0%) (Fig. 4). Using SMD, we pooled data from trials that reported base excess as a surrogate for serum bicarbonate, and the result showed a reduction in SMD of −3.98 meq/L (95% CI −5.47 to −2.49; *I*^2^ = 94%) (Additional file 2: Figure S7).

#### pH

**Fig. 5:**
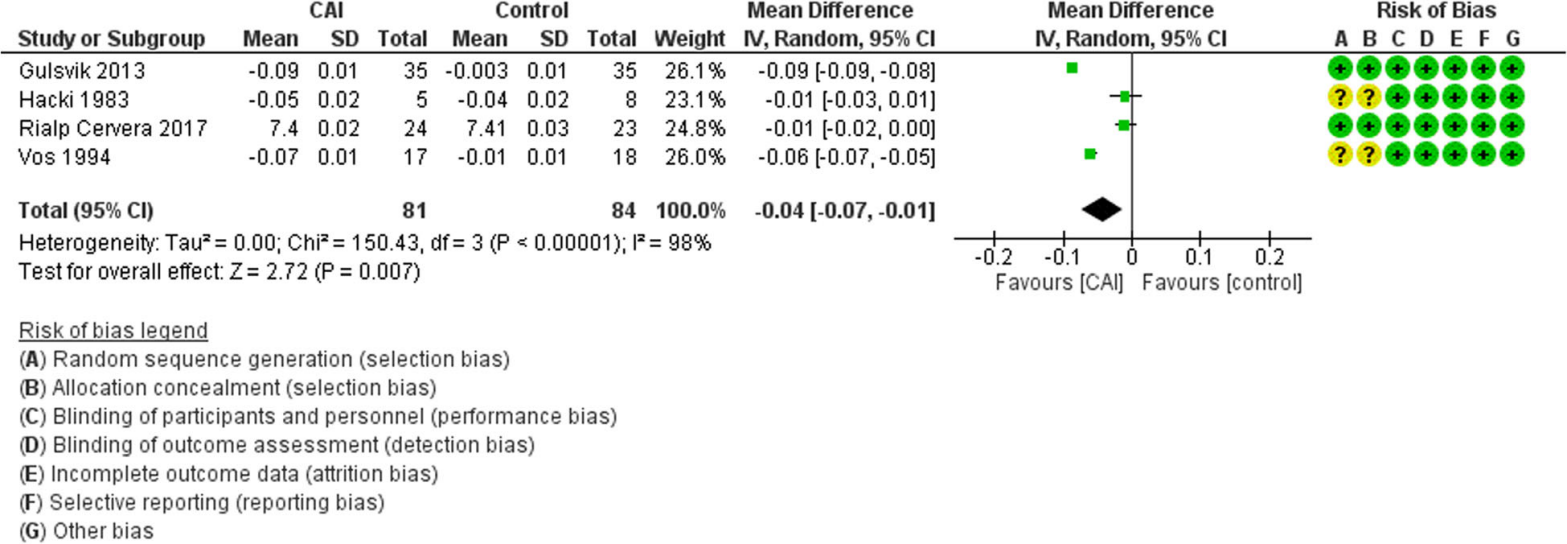
Forest plot for the effect of carbonic anhydrase inhibitors (CAI) vs control on pH. CI confidence interval, IV Inverse variance, SD standard deviation

The main analysis excluded Faisy et al. because they reported the daily mean change in pH. The results showed a mean reduction in pH of 0.04 in the CAI group (95% CI −0.07 to −0.01; *I*^2^ = 98%) (Fig. 5). The result did not change substantially when Faisy et al. was incorporated using SMD (Additional file 2: Figure S8).

#### Adverse events

**Fig. 6:**
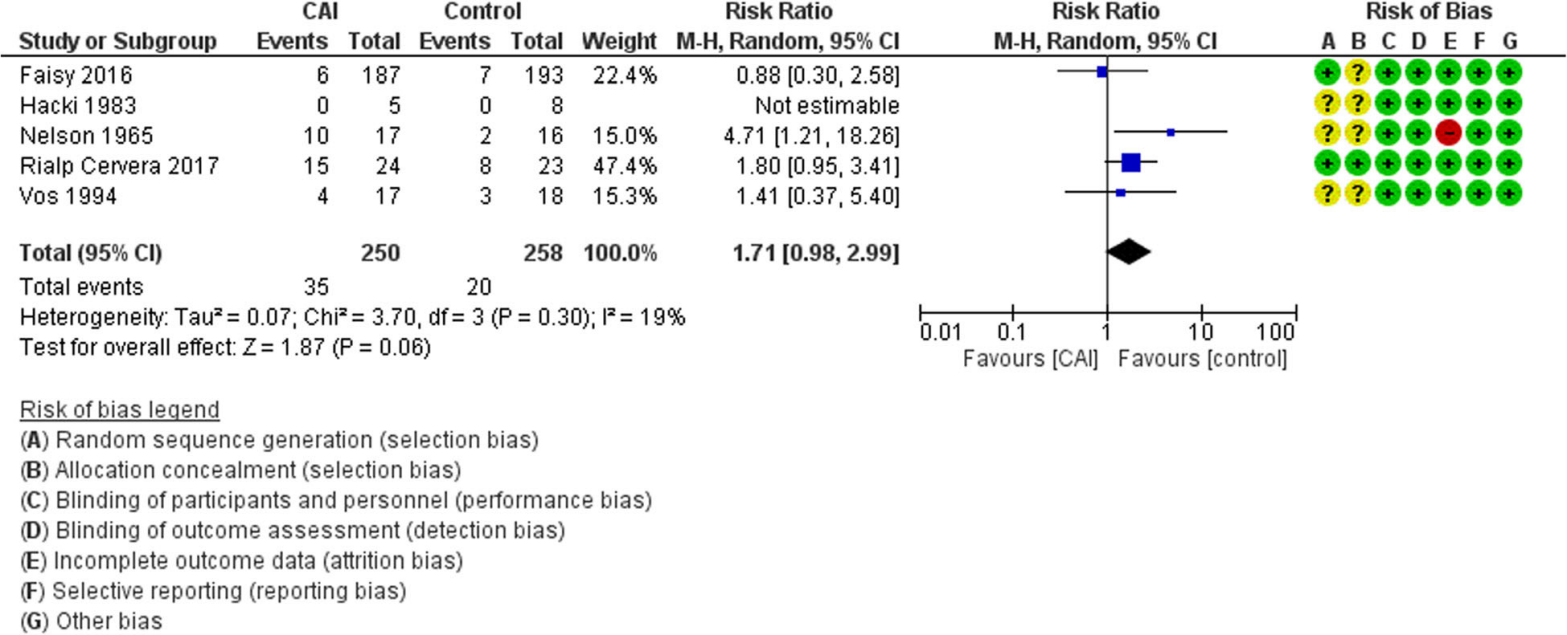
Forest plot for the effect of carbonic anhydrase inhibitors (CAI) vs control on adverse events. CI confidence interval, M-H Mantel-Haenszel, SD standard deviation

The analysis from the five trials that reported on adverse outcomes showed an increased risk of adverse events in the CAI group (RR 1.71, 95% CI 0.98 to 2.99; *I*^2^ = 19%) (Fig. 6). Certainty of evidence was judged to be moderate due to imprecision (Table 3).

This result was mainly driven by increased incidence of mild side effects such as vertigo, paresthesia, nausea, vomiting, headache, skin rash, and abdominal discomfort in Nelson and Wallace and Vos et al. [12, 29], and increased incidence of hypokalemia and serum bicarbonate < 23 mmol/L in the acetazolamide group in Rialp Cervera et al. [8]. In the study of Hacki et al., the authors reported that no side effects or severe metabolic acidosis were noted in the two groups [15].

On the other hand, there was no increased incidence of serious adverse reactions in the CAI group in the study of Faisy et al., as defined by events that required intensive care procedures and/or surgery, and events that prolonged hospitalization or resulted in persistent or major disability/incapacity, and in Rialp Cervera et al. as defined by serum creatinine > 2.5 g/dL, bilirubin > 3.5 mg/dL, prothrombin activity < 40%, leukocyte count < 4 × 10^9^/L, platelets < 150 × 10^9^/L, appearance of seizures, or severe allergic reaction [6, 8].

#### Subgroup and sensitivity analyses

Pre-planned subgroup and sensitivity analyses were not possible because of the limited number of included trials.

## Discussion

In summary, we did not find definitive results for the effects of CAI therapy on clinically important outcomes such as mortality and duration of hospital stay in patients with respiratory failure and metabolic alkalosis. The results suggest that CAI therapy may decrease the duration of mechanical ventilation. There was a trend towards increased incidence of adverse events in the CAI group; however, most of these adverse events were mild.

On the other hand, the results suggest that CAI therapy has favorable effects on arterial blood gas parameters (PaCO_2_, PaO_2_, bicarbonate and pH), with decreased PaCO_2_, increased PaO_2_, and, as expected, decreased bicarbonate and pH levels.

The main strength of the current review is the use of rigorous systematic review methods. Also, inclusion criteria were restricted to well-designed RCTs with an overall low risk of bias.

The limitations of the current systematic review relate to those of the included studies, mainly their limited number. In addition, some of the included studies did not report all the data required to include them in the meta-analyses. For example, Nelson and Wallace did not provide SDs, and Faisy et al. reported medians and IQRs but not means and SDs. Furthermore, most trials used acetazolamide, and most patients had COPD, and thus our results may not be applicable to CAIs other than acetazolamide and in respiratory conditions other than COPD.

A previously published systematic review focusing on patients with COPD and hypercapnic respiratory failure included four trials with 84 participants. That study reported that acetazolamide therapy significantly decreased pH and serum bicarbonate and significantly increased PaO_2_, and led to a small decrease in PaCO_2_. The study reported those results as not statistically significant [5]. We have excluded two of the trials included in that review (Skatrud and Dempsey [31] and Wagenaar et al. [32]) because they did not meet our eligibility criteria. Skatrud and Dempsey reported outcomes exclusively in patients deemed as “correctors” to acetazolamide, and in Wagenaar et al. the arms comparing acetazolamide with placebo were not randomized [31, 32]. Furthermore, none of the trials included in that previous review assessed clinically important outcomes. The present review included six trials with a total of 564 participants, and it provides higher certainty evidence for the favorable effects of CAI therapy on blood gases parameters. In addition, two trials included in our review assessed clinically important outcomes.

Although the results of the pooled analysis concerning mortality and duration of hospital stay are not conclusive, they suggest a possible decrease in the duration of mechanical ventilation in the CAI group compared with placebo. This clinically important outcome should be confirmed in future larger randomized clinical trials.

## Conclusion

The present systematic review demonstrates that carbonic anhydrase inhibitors are associated with favorable blood gas parameters in patients with respiratory failure and metabolic alkalosis, but did not provide conclusive results for clinically important outcomes. Future well-designed and large randomized trials should investigate the effect of carbonic anhydrase inhibitors on these outcomes, particularly mortality, duration of hospital stay, and duration of mechanical ventilation.

## Additional files

Additional file 1: Details of the methods. (PDF 41 kb)

Additional file 2: **Table S1.** MEDLINE search strategy. **Table S2.** EMBASE search strategy. **Table S3.** SCOPUS search strategy. **Table S4** Cochrane CENTRAL search strategy. **Table S5.** Funding and conflict of interest of authors in the included trials. **Figure S1.** Risk of bias summary. **Figure S2.** Forest plot for the effect of CAI vs control on mortality. **Figure S3.** Forest plot for the effect of CAI vs control on duration of hospital stay. **Figure S4.** Forest plot for the effect of CAI vs control on duration of mechanical ventilation. **Figure S5.** Forest plot for the effect of CAI vs control on PaCO_2_ using standardized mean difference. **Figure S6.** Forest plot for the effect of CAI vs control on PaO_2_ using standardized mean difference. **Figure S7.** Forest plot for the effect of CAI vs control on serum bicarbonate using standardized mean difference. **Figure S8.** Forest plot for the effect of CAI vs control on pH using standardized mean difference. (PDF 338 kb)

## Abbreviations

CAI: Carbonic anhydrase inhibitors
CI: Confidence interval
COPD: Chronic obstructive pulmonary disease
IQR: Interquartile range
MD: Mean difference
MV: Mechanical ventilation
NIPPV: Noninvasive positive-pressure ventilation
RCT: Randomized controlled trial
RR: Risk ratio
SD: Standard deviation
SMD: Standardized mean difference
SoF: Summary of findings
