# Supplementary material for: Carbonic anhydrase inhibitors in patients with respiratory failure and metabolic alkalosis: a systematic review and meta-analysis of randomized controlled trials
Source: Crit Care. 2018 Oct 29;22:275. doi: 10.1186/s13054-018-2207-6 (PMC6205780; doi:10.1186/s13054-018-2207-6)
Supplement: Supplementary file 1 — Details of the methods. (PDF 41 kb) [file 13054_2018_2207_MOESM1_ESM.pdf]

Detailed Methods

Protocol and registration

The review protocol is registered in PROSPERO (PROSPERO 2017 CRD42017072501 Available from: http://www.crd.york.ac.uk/PROSPERO/display_record.php?ID=CRD42017072501)

Eligibility criteria

RCTs were included if they recruited patients with respiratory failure and concurrent metabolic alkalosis (as defined by the individual trial), including patients on MV or NIPPV. In addition, the trial should have compared CAI to either placebo or usual care. All co-interventions should have been similar for the two comparison groups.

The pre-specified primary outcomes of interest were duration of hospital stay, duration of MV or NIPPV, mortality and adverse events. Secondary outcomes included blood gases parameters: $PaCO_2$, PO_2, HCO_3, and PH.

Search strategy

We searched the following electronic databases from inception to August 2017: the Cochrane Central Register of Controlled Trials (CENTRAL), MEDLINE, EMBASE, and SCOPUS. Tables 1 to 4 in additional file detail the electronic search strategy. There were no language or date restrictions. We also screened the reference lists of included trials and identified related systematic reviews.

Selection process

Teams of two review authors (BT, CN and HI, MO) screened in duplicate and independently the abstract and title of every record captured by the searches for potential eligibility. We retrieved the full texts for all citations judged as potentially eligible by at least one of the reviewers.

The teams of two reviewers then assessed in duplicate and independently the full texts for eligibility using a standardized and pilot tested screening form. They then compared their results and resolved any disagreements by consensus and, when unsuccessful, with the help of a third reviewer (SM or PBK). Before starting the selection process, BT, HI, CN, and MO conducted calibration exercises to ensure the validity of the selection process.

A PRISMA (preferred reporting items for systematic reviews and meta-analyses) flow-chart was used to summarize the results of the selection process.(9)

Data extraction

For each included study, the teams of two reviewers independently and in duplicate abstracted relevant information using standardized data extraction forms. They then compared their results and resolved any disagreements by discussion and, when unsuccessful, with the help of a third reviewer.

We extracted information about the study design; the clinical characteristics of the trial (population, intervention, comparator, and outcomes); funding, and conflicts of interest of authors.

Assessment of risk of bias

The two teams of reviewers assessed the risk of bias of each included trial independently and in duplicate. Disagreements were resolved by consensus and, when unsuccessful, with the help of a third reviewer.

Risk of bias was assessed using The Cochrane Collaboration's Risk of Bias tool.(10, 11) The following criteria were used: random sequence generation (selection bias), allocation concealment (selection bias), blinding of participants, providers, data collectors, outcome adjudicators, and data analysts (performance bias and detection bias), incomplete outcome data (attrition bias), selective outcome reporting (reporting bias), other bias (including early stopping for benefit).

We judged Risk of bias criteria as 'low risk', 'high risk' or 'unclear risk' and evaluated individual bias items as described in the *Cochrane Handbook for Systematic Reviews of Interventions*.(10)

Data analysis

For dichotomous data, we used risk ratio (RR) or hazard ratio (HR) with 95% confidence intervals (CI). For continuous outcomes data, we used, whenever possible, the mean change score from baseline to follow-up for each intervention group.

One of the of the included trials (Nelson 1965) did not report standard deviations (SD) in the assessment of the outcomes PaCO2 and serum bicarbonate.(12) Therefore, we used the median SD from the other

included trials that reported SDs for these outcomes, as described in Furukawa 2006.(13) In another trial (Faisy 2016),(14) the authors did not report means and SDs, so these were extrapolated respectively from the reported medians (mean = median) and interquartile ranges (IQR) (SD= IQR/1.35).(10) In Hacki 1983, outcomes data were extracted from a graph in the report using the WebPlotDigitizer tool.(15, 16)

We pooled data using the random-effects model for the primary meta-analyses.(17) Heterogeneity (inconsistency) between study results was assessed by visual inspection of the forest plots and by using the I^2 statistic. We considered an I^2 value of 50% or more to indicate a considerable level of heterogeneity.(10) In order to explain any heterogeneity, we planned to conduct subgroup analyses based on the following variables: specific type and dose of CAI, etiology of respiratory failure, spontaneously breathing patients or on MV or NIPPV, and severity of metabolic alkalosis.

We also planned to perform sensitivity analyses to explore the influence of the following factors (when applicable) on pooled effect sizes: restricting the analyses to studies with low risk of bias, restricting the analyses to studies with longer follow-up, and assessing the impact of missing data. (18-21).

Assessment of certainty of the evidence

Certainty of the evidence for each outcome was assessed using the Grading of Recommendations Assessment, Development and Evaluation (GRADE) approach.(22) The approach classifies the certainty of evidence in into four categories: high, moderate, low and very low. It takes into account the following factors: risk of bias,(23) imprecision, inconsistency,(24) indirectness,(25) and publication bias.(26) We developed a Summary of Findings (SoF) table using the GRADEpro/GDT tool.(27)
