# Supplementary material for: Carbonic anhydrase inhibitors in patients with respiratory failure and metabolic alkalosis: a systematic review and meta-analysis of randomized controlled trials
Source: Crit Care. 2018 Oct 29;22:275. doi: 10.1186/s13054-018-2207-6 (PMC6205780; doi:10.1186/s13054-018-2207-6)
Supplement: Supplementary file 2 — Table S1. MEDLINE search strategy. Table S2. EMBASE search strategy. Table S3. SCOPUS search strategy. Table S4 Cochrane CENTRAL search strategy. Table S5. Funding and conflict of interest of authors in the included trials. Figure S1. Risk of bias summary. Figure S2. Forest plot for the effect of CAI vs control on mortality. Figure S3. Forest plot for the effect of CAI vs control on duration of hospital stay. Figure S4. Forest plot for the effect of CAI vs control on duration of mechanical ventilation. Figure S5. Forest plot for the effect of CAI vs control on PaCO2 using standardized mean difference. Figure S6. Forest plot for the effect of CAI vs control on PaO2 using standardized mean difference. Figure S7. Forest plot for the effect of CAI vs control on serum bicarbonate using standardized mean difference. Figure S8. Forest plot for the effect of CAI vs control on pH using standardized mean difference. (PDF 338 kb) [file 13054_2018_2207_MOESM2_ESM.pdf]

Table 1: Medline search strategy

Medline	
**#**	**Searches**
**1**	exp Alkalosis/
**2**	(alkalosis or alkaloses).mp. [mp=title, abstract, original title, name of substance word, subject heading word, keyword heading word, protocol supplementary concept word, rare disease supplementary concept word, unique identifier, synonyms] (7954)<br>51 (electrolyte adj2 imbalance).mp. [mp=title, abstract, original title, name of substance word, subject heading word, keyword heading word, protocol supplementary concept word, rare disease supplementary concept word, unique identifier, synonyms]
**3**	exp Hypercapnia/
**4**	hypercapni*.mp.
**5**	hypoxi*.mp.
**6**	exp Hypoxia/
**7**	(anoxemi* or anoxi* or (oxygen adj2 deficienc*) or hypoxemi*).mp.
**8**	exp Ventilators, Mechanical/
**9**	(ventilator* or respirator* or (artificial adj2 respiration*)).mp.
**10**	exp Positive-Pressure Respiration/
**11**	((positive adj2 pressure adj2 (ventilation* or respiration*)) or (positive adj2 expiratory adj2 pressure)).mp.
**12**	exp Intermittent Positive-Pressure Ventilation/ or exp Noninvasive Ventilation/
**13**	NIPPV.mp.
**14**	BiPAP.mp.
**15**	(ippv or (inspiratory adj2 positive adj2 pressure adj2 (ventilation or ventilator)) or aprv or CPAP or ncpap or (positive adj2 airway adj2 pressure)).mp. [mp=title, abstract, original title, name of substance word, subject heading word, keyword heading word, protocol supplementary concept word, rare disease supplementary concept word, unique identifier, synonyms]
**16**	((respiratory or ventilator) adj2 (failure or insufficiency or depression)).mp.
**17**	exp Respiratory Insufficiency/
**18**	(CO2 adj2 retention).mp.
**19**	((respiratory or bronchial* or pulmonary or lung or (silo adj2 filller) or (urbach adj2 wiethe) or tracheal or mediastinal or pleural or otolaryngologic* or laryngeal or pharyngeal or gilchrist* or larynx) adj2 disease*).mp. [mp=title, abstract, original title, name of substance word, subject heading word, keyword heading word, protocol supplementary concept word, rare disease supplementary concept word, unique identifier, synonyms]
**20**	(asthma* or bronchospasm* or ((aspirin or nsaid or kartagener* or siewert or (williams adj2 campbell) or (persistent adj2 fetal adj2 circulation*) or hepatopulmonary or caplan or goodpasture* or pancoast* or macleod or (swyer adj2 jame*) or mendelson or aspiration or brock* or loeffler or (respiratory adj2 distress) or chest or scimitar or pickwickian) adj2 syndrome*) or bronchiectas* or dextrocardia or sinusitis or (ciliary adj2 dyskinesia) or ((pulmonary or lung) adj2 (cancer or neoplasm*)) or bronchiolitis* or bronchitis* or boop or pneumonia* or chondromalacia* or tracheobronchomegal* or (cilia* adj2 (dyskinesia* or motility)) or bronchiectasis or granuloma*).mp. [mp=title,

	abstract, original title, name of substance word, subject heading word, keyword heading word, protocol supplementary concept word, rare disease supplementary concept word, unique identifier, synonyms]
**21**	(((laryngeal or larynx) adj2 (perichondriti* or edema* or cancer or neoplasm* or avulsion*or transection*or injur*or chondromalacia* or stenos*)) or laryngiti* or croup or laryngocele* or laryngomalacia* or supraglottiti* or (subglottic adj2 stenos*)).mp. [mp=title, abstract, original title, name of substance word, subject heading word, keyword heading word, protocol supplementary concept word, rare disease supplementary concept word, unique identifier, synonyms]
**22**	(tuberculos* or (vocal adj2 cord* adj2 dysfunction*) or (paradoxical adj2 vocal adj2 fold) or laryngismus or laryngospasm* or (laryngeal adj2 spasm*) or aphonia* or dysphonia*).mp. or (exp Alkalosis/ adj2 antitrypsin.mp. adj2 defecienc*.mp.) or (adenomatoid adj2 malformation*).mp. or ((cystic or pulmonary or radiation) adj2 fibrosis).mp. or mucoviscidosis.mp. or hemoptys*.mp. or (pulmonary adj2 hypertension*).mp. or (acd adj2 mpv).mp. or (alveolar adj2 capillary adj2 dysplasia*).mp. or (ductus adj2 arteriosus).mp. or (hht adj2 pph1).mp. or ((lung or pulmonary) adj2 abscess*).mp. or blastomycos*.mp. or pneumocyst*.mp. or aspergillosis.mp. or alveoliti*.mp. or ((fancier* or farmer or mushroom or black or coal or brown) adj2 lung*).mp. [mp=title, abstract, original title, name of substance word, subject heading word, keyword heading word, protocol supplementary concept word, rare disease supplementary concept word, unique identifier, synonyms]
**23**	(trichosporonos* or (anti adj2 gbm) or (((anti adj2 glomerular) or (hand adj2 schuller adj2 christian) or (letterer adj2 swie) or (hashimoto adj2 pritzge) or beryllium or (hamman adj2 rich)) adj2 disease*) or (lung adj2 purpra adj2 nephritis) or langerhan* or histocytos* or reticulendothelios* or bagassosis or pneumoconios* or anthracos* or anthracosilicos* or asbestos* or beryllios* or byssinos* or sideros* or silicos* or silicotuberculos* or myelobastin or bronchitis or copd or coad or (chronic adj2 airflow adj2 obstruction*) or emphysema* or (pulmonary adj2 (infection* or cyst or hydatid* or edema* or granuloma* or pseudotumor* or collapse or embolism* or eosinophili* or infarction)) or (lung adj2 (injur* or carcinoma* or hyperlucent* or collapse or wet*)) or (meconium adj2 aspiration) or (bronchopulmonary adj2 dysplasia) or bronchogeni* or (pulmonary adj2 nodule*) or (respiratory adj2 aspiration*) or atelectas*).mp.
**24**	(hyalinosis or (hyaline adj2 membrane adj2 disease*) or tachypnea or anomal* or tapvr or (total adj2 anomalous adj2 pulmonary adj2 venous adj2 return) or (pulmonary adj2 lesion*) or pneumonectomy or (pleural adj2 disease*) or empyema* or hemothrax or (pleural adj2 (effusion* or neoplasm*)) or thoracentesis or pleuri* or pneumothorax or tuberculous or hypoxia* or (mountain adj2 sickness) or apnea* or (cheyne adj2 stokes adj2 respiration*) or breathlessness*).mp. [mp=title, abstract, original title, name of substance word, subject heading word, keyword heading word, protocol supplementary concept word, rare disease supplementary concept word, unique identifier, synonyms]
**25**	((breath adj2 shortness) or dyspnea* or hoarseness*).mp. [mp=title, abstract, original title, name of substance word, subject heading word, keyword heading word, protocol supplementary concept word, rare disease supplementary concept word, unique identifier, synonyms]
**26**	(hyperventilation* or (mouth adj2 breathing*) or (respiratory adj2 depression) or choking or ((airway or nasal) adj2 obstruction*) or hards).mp. [mp=title, abstract,

	original title, name of substance word, subject heading word, keyword heading word, protocol supplementary concept word, rare disease supplementary concept word, unique identifier, synonyms]
**27**	((hantavirus adj2 associated adj2 respiratory adj2 distress adj2 syndrome*) or hypoventilation* or (sequestration* adj2 pulmonary) or (choanal adj2 atresia*) or (lung adj2 malformation adj2 adenomatoid*) or bronchiectasis or whooping).mp.
**28**	(tracheitis or pertuss*).mp. [mp=title, abstract, original title, name of substance word, subject heading word, keyword heading word, protocol supplementary concept word, rare disease supplementary concept word, unique identifier, synonyms]
**29**	exp respiratory tract diseases/ or exp bronchial diseases/ or exp ciliary motility disorders/ or exp granuloma, respiratory tract/ or exp laryngeal diseases/ or exp lung diseases/ or nose diseases/ or exp pleural diseases/ or exp respiration disorders/ or acute chest syndrome/ or exp apnea/ or cheyne-stokes respiration/ or exp dyspnea/ or exp hoarseness/ or exp hyperventilation/ or meconium aspiration syndrome/ or mouth breathing/ or exp respiratory aspiration/ or exp respiratory distress syndrome, adult/ or exp respiratory distress syndrome, newborn/ or exp respiratory insufficiency/ or acidosis, respiratory/ or exp airway obstruction/ or granuloma, laryngeal/ or hantavirus pulmonary syndrome/ or exp hypoventilation/ or infantile apparent life-threatening event/ or positive-pressure respiration, intrinsic/ or respiratory paralysis/ or sarcoglycanopathies/ or tachypnea/ or vocal cord dysfunction/ or exp respiratory hypersensitivity/ or exp respiratory system abnormalities/ or exp respiratory tract fistula/ or exp respiratory tract infections/ or exp respiratory tract neoplasms/ or exp thoracic diseases/ or exp tracheal diseases/
**30**	(acetazolamide* or (carbon* adj2 (dehydratase or anhydrase) adj2 inhibitor*) or (carboxyanhydrase adj2 inhibitor*) or acetadiazol or acetazolam* or akzol or (ak adj2 zol) or defiltran or diacarb or diamox or diuramide or edemox or glauconox or glaupax or (huma adj2 zolamide) or benzolamide or (cl adj2 "11366") or sulfonamide* or thiadiazole* or daranide or dichlofenamid* or dichlorphenamid* or glauconide or methazolamide* or (n adj2 mrthylacetazolamide)).mp.
**31**	carbonic anhydrase inhibitors/ or exp acetazolamide/ or exp benzolamide/ or exp dichlorphenamide/ or exp methazolamide/
**32**	or/1-29
**33**	or/30-31
**34**	((randomized controlled trial or controlled clinical trial).pt. or randomized.ab. or placebo.ab. or drug therapy.fs. or randomly.ab. or trial.ab. or groups.ab.) not (exp animals/ not humans.sh.)
**35**	32 and 33 and 34

Table 2: EMBASE search strategy

**EMBASE**	
**#**	**Searches**
**1**	'alkalosis'/exp OR 'alkalosis':ti,ab,kw OR 'alkaloses':ti,ab,kw OR 'electrolyte disturbance'/exp OR (('electrolyte' NEAR/2 'imbalance'):ti,ab,kw) OR 'hypercapnia'/exp OR 'hypercapni*':ti,ab,kw OR 'hypoxia'/exp OR 'hypoxi*':ti,ab,kw OR 'anoxemi*':ti,ab,kw OR 'anoxi*':ti,ab,kw OR (('oxygen' NEAR/2 'deficienc*'):ti,ab,kw)

	OR 'hypoxemi*':ti,ab,kw OR 'mechanical ventilator'/exp OR 'ventilator*':ti,ab,kw OR 'respirator*':ti,ab,kw OR (('artificial' NEAR/2 'respiration*'):ti,ab,kw) OR (('positive' NEAR/2 'pressure' NEAR/2 '(ventilation*'):ti,ab,kw) OR 'respiration*)':ti,ab,kw OR (('positive' NEAR/2 'expiratory' NEAR/2 'pressure'):ti,ab,kw) OR 'intermittent positive pressure ventilation'/exp OR 'noninvasive ventilation'/exp OR 'nippv':ti,ab,kw OR 'bipap':ti,ab,kw OR 'ippv':ti,ab,kw OR (('(inspiratory' NEAR/2 'positive' NEAR/2 'pressure' NEAR/2 '(ventilation'):ti,ab,kw) OR 'ventilator))':ti,ab,kw OR 'aprv':ti,ab,kw OR 'cpap':ti,ab,kw OR 'ncpap':ti,ab,kw OR (('(positive' NEAR/2 'airway' NEAR/2 'pressure)'):ti,ab,kw) OR (('ventilator)' NEAR/2 '(failure'):ti,ab,kw) OR 'insufficiency':ti,ab,kw OR 'depression)':ti,ab,kw OR 'respiratory insufficiency'/exp OR (('co2' NEAR/2 'retention'):ti,ab,kw) OR '(respiratory':ti,ab,kw OR 'bronchial*':ti,ab,kw OR 'lung':ti,ab,kw OR (('(silo' NEAR/2 'filller)'):ti,ab,kw) OR (('(urbach' NEAR/2 'wiethe)'):ti,ab,kw) OR 'tracheal':ti,ab,kw OR 'mediastinal':ti,ab,kw OR 'pleural':ti,ab,kw OR 'otolaryngologic*':ti,ab,kw OR 'laryngeal':ti,ab,kw OR 'pharyngeal':ti,ab,kw OR 'gilchrist*':ti,ab,kw OR (('larynx)' NEAR/2 'disease*'):ti,ab,kw) OR 'asthma*':ti,ab,kw OR 'bronchospasm*':ti,ab,kw OR '((aspirin':ti,ab,kw OR 'nsaid':ti,ab,kw OR 'kartagener*':ti,ab,kw OR 'siewert':ti,ab,kw OR (('(williams' NEAR/2 'campbell)'):ti,ab,kw) OR (('(persistent' NEAR/2 'fetal' NEAR/2 'circulation*)'):ti,ab,kw) OR 'hepatopulmonary':ti,ab,kw OR 'caplan':ti,ab,kw OR 'goodpasture*':ti,ab,kw OR 'pancoast*':ti,ab,kw OR 'macleod':ti,ab,kw OR (('(swyer' NEAR/2 'jame*)'):ti,ab,kw) OR 'mendelson':ti,ab,kw OR 'aspiration':ti,ab,kw OR 'brock*':ti,ab,kw OR 'loeffler':ti,ab,kw OR (('(respiratory' NEAR/2 'distress)'):ti,ab,kw) OR 'chest':ti,ab,kw OR 'scimitar':ti,ab,kw OR (('pickwickian)' NEAR/2 'syndrome*)'):ti,ab,kw) OR 'bronchiectas*':ti,ab,kw OR 'dextrocardia':ti,ab,kw OR 'sinusitis':ti,ab,kw OR (('(ciliary' NEAR/2 'dyskinesia)'):ti,ab,kw) OR '((pulmonary':ti,ab,kw OR (('lung)' NEAR/2 '(cancer'):ti,ab,kw) OR 'bronchiolitis*':ti,ab,kw OR 'bronchitis*':ti,ab,kw OR 'boop':ti,ab,kw OR 'pneumonia*':ti,ab,kw OR 'chondromalacia*':ti,ab,kw OR 'tracheobronchomegal*':ti,ab,kw OR (('(cilia*' NEAR/2 '(dyskinesia*'):ti,ab,kw) OR 'motility))':ti,ab,kw OR '((laryngeal':ti,ab,kw OR (('larynx)' NEAR/2 '(perichondriti*'):ti,ab,kw) OR 'cancer':ti,ab,kw OR 'neoplasm*':ti,ab,kw OR 'avulsion*or transection*or injur*or chondromalacia*':ti,ab,kw OR 'stenos*))':ti,ab,kw OR 'laryngiti*':ti,ab,kw OR 'croup':ti,ab,kw OR 'laryngocele*':ti,ab,kw OR 'laryngomalacia*':ti,ab,kw OR 'supraglottiti*':ti,ab,kw OR (('(subglottic' NEAR/2 'stenos*)'):ti,ab,kw) OR 'tuberculos*':ti,ab,kw OR (('(vocal' NEAR/2 'cord*' NEAR/2 'dysfunction*)'):ti,ab,kw) OR (('(paradoxical' NEAR/2 'vocal' NEAR/2 'fold)'):ti,ab,kw) OR 'laryngismus':ti,ab,kw OR 'laryngospasm*':ti,ab,kw OR (('(laryngeal' NEAR/2 'spasm*)'):ti,ab,kw) OR 'aphonia*':ti,ab,kw OR 'dysphonia*)':ti,ab,kw OR (('(antitrypsin' NEAR/2 'defecienc*)'):ti,ab,kw) OR (('(adenomatoid' NEAR/2 'malformation*)'):ti,ab,kw) OR '((cystic':ti,ab,kw OR 'pulmonary':ti,ab,kw OR (('radiation)' NEAR/2 'fibrosis)'):ti,ab,kw) OR 'mucoviscidosis':ti,ab,kw OR 'hemoptys*':ti,ab,kw OR (('(pulmonary' NEAR/2 'hypertension*)'):ti,ab,kw) OR (('(acd' NEAR/2 'mpv)'):ti,ab,kw) OR (('(alveolar' NEAR/2 'capillary' NEAR/2 'dysplasia*)'):ti,ab,kw) OR (('(ductus' NEAR/2 'arteriosus)'):ti,ab,kw) OR (('(hht' NEAR/2 'pph1)'):ti,ab,kw) OR '((lung':ti,ab,kw OR (('pulmonary)' NEAR/2 'abscess*)'):ti,ab,kw) OR 'blastomycos*':ti,ab,kw OR 'pneumocyst*':ti,ab,kw OR 'aspergillosis':ti,ab,kw OR 'alveoliti*':ti,ab,kw OR '((fancier*':ti,ab,kw OR

	'farmer':ti,ab,kw OR 'mushroom':ti,ab,kw OR 'black':ti,ab,kw OR 'coal':ti,ab,kw OR (('brown)' NEAR/2 'lung*'):ti,ab,kw) OR 'trichosporonos*':ti,ab,kw OR (('(anti' NEAR/2 'gbm)'):ti,ab,kw) OR (('(((anti' NEAR/2 'glomerular)'):ti,ab,kw) OR (('(hand' NEAR/2 'schuller' NEAR/2 'christian)'):ti,ab,kw) OR (('(letterer' NEAR/2 'swie)'):ti,ab,kw) OR (('(hashimoto' NEAR/2 'pritzge)'):ti,ab,kw) OR 'beryllium':ti,ab,kw OR (('(hamman' NEAR/2 'rich))' NEAR/2 'disease*)'):ti,ab,kw) OR (('(lung' NEAR/2 'purpra' NEAR/2 'nephritis)'):ti,ab,kw) OR 'langerhan*':ti,ab,kw OR 'histocytos*':ti,ab,kw OR 'reticulendothelios*':ti,ab,kw OR 'bagassosis':ti,ab,kw OR 'pneumoconios*':ti,ab,kw OR 'anthracos*':ti,ab,kw OR 'anthracosilicos*':ti,ab,kw OR 'asbestos*':ti,ab,kw OR 'beryllios*':ti,ab,kw OR 'byssinos*':ti,ab,kw OR 'sideros*':ti,ab,kw OR 'silicos*':ti,ab,kw OR 'silicotuberculos*':ti,ab,kw OR 'myelobastin':ti,ab,kw OR 'bronchitis':ti,ab,kw OR 'copd':ti,ab,kw OR 'coad':ti,ab,kw OR (('(chronic' NEAR/2 'airflow' NEAR/2 'obstruction*)'):ti,ab,kw) OR 'emphysema*':ti,ab,kw OR (('(pulmonary' NEAR/2 '(infection*'):ti,ab,kw) OR 'cyst':ti,ab,kw OR 'hydatid*':ti,ab,kw OR 'edema*':ti,ab,kw OR 'granuloma*':ti,ab,kw OR 'pseudotumor*':ti,ab,kw OR 'embolism*':ti,ab,kw OR 'eosinophili*':ti,ab,kw OR 'infarction))':ti,ab,kw OR (('(lung' NEAR/2 '(injur*'):ti,ab,kw) OR 'carcinoma*':ti,ab,kw OR 'hyperlucent*':ti,ab,kw OR 'collapse':ti,ab,kw OR 'wet*))':ti,ab,kw OR (('(meconium' NEAR/2 'aspiration)'):ti,ab,kw) OR (('(bronchopulmonary' NEAR/2 'dysplasia)'):ti,ab,kw) OR 'bronchogeni*':ti,ab,kw OR (('(pulmonary' NEAR/2 'nodule*)'):ti,ab,kw) OR (('(respiratory' NEAR/2 'aspiration*)'):ti,ab,kw) OR 'atelectas*':ti,ab,kw OR 'hyalinosis':ti,ab,kw OR (('(hyaline' NEAR/2 'membrane' NEAR/2 'disease*)'):ti,ab,kw) OR 'tachypnea':ti,ab,kw OR 'anomal*':ti,ab,kw OR 'tapvr':ti,ab,kw OR (('(total' NEAR/2 'anomalous' NEAR/2 'pulmonary' NEAR/2 'venous' NEAR/2 'return)'):ti,ab,kw) OR (('(pulmonary' NEAR/2 'lesion*)'):ti,ab,kw) OR 'pneumonectomy':ti,ab,kw OR (('(pleural' NEAR/2 'disease*)'):ti,ab,kw) OR 'empyema*':ti,ab,kw OR 'hemothrax':ti,ab,kw OR (('(pleural' NEAR/2 '(effusion*'):ti,ab,kw) OR 'neoplasm*))':ti,ab,kw OR 'thoracentesis':ti,ab,kw OR 'pleuri*':ti,ab,kw OR 'pneumothorax':ti,ab,kw OR 'tuberculous':ti,ab,kw OR 'hypoxia*':ti,ab,kw OR (('(mountain' NEAR/2 'sickness)'):ti,ab,kw) OR 'apnea*':ti,ab,kw OR (('(cheyne' NEAR/2 'stokes' NEAR/2 'respiration*)'):ti,ab,kw) OR 'breathlessness*':ti,ab,kw OR (('(breath' NEAR/2 'shortness)'):ti,ab,kw) OR 'dyspnea*':ti,ab,kw OR 'hoarseness*':ti,ab,kw OR 'hyperventilation*':ti,ab,kw OR (('(mouth' NEAR/2 'breathing*)'):ti,ab,kw) OR (('(respiratory' NEAR/2 'depression)'):ti,ab,kw) OR 'choking':ti,ab,kw OR '((airway':ti,ab,kw OR (('nasal)' NEAR/2 'obstruction*)'):ti,ab,kw) OR 'hards':ti,ab,kw OR (('(hantavirus' NEAR/2 'associated' NEAR/2 'respiratory' NEAR/2 'distress' NEAR/2 'syndrome*)'):ti,ab,kw) OR 'hypoventilation*':ti,ab,kw OR (('(sequestration*' NEAR/2 'pulmonary)'):ti,ab,kw) OR (('(choanal' NEAR/2 'atresia*)'):ti,ab,kw) OR (('(lung' NEAR/2 'malformation' NEAR/2 'adenomatoid*)'):ti,ab,kw) OR 'bronchiectasis':ti,ab,kw OR 'whooping':ti,ab,kw OR 'tracheitis':ti,ab,kw OR 'pertuss*':ti,ab,kw
**2**	'respiratory tract disease'/exp OR 'bronchus disease'/exp OR 'ciliary motility disorders'/exp OR 'granuloma'/exp OR 'larynx disorder'/exp OR 'lung disease'/exp OR 'nose disease'/exp OR 'pleura disease'/exp OR 'breathing disorder'/exp OR 'apnea'/exp OR 'dyspnea'/exp OR 'hoarseness'/exp OR 'meconium aspiration'/exp OR 'mouth breathing'/exp OR 'respiratory distress syndrome'/exp OR 'respiratory failure'/exp OR

	'airway obstruction'/exp OR 'diaphragm paralysis'/exp OR 'sarcoglycanopathy'/exp OR 'tachypnea'/exp OR 'vocal cord disorder'/exp OR 'positive end expiratory pressure'/exp OR 'respiratory tract allergy'/exp OR 'respiratory tract malformation'/exp OR 'respiratory tract fistula'/exp OR 'respiratory tract infection'/exp OR 'respiratory tract tumor'/exp OR 'thorax disease'/exp OR 'pneumonia'/exp OR 'trachea disease'/exp
**3**	#1 OR #2
**4**	'acetazolamide*':ti,ab,kw OR (('carbon*' NEAR/2 'dehydratase' NEAR/2 'inhibitor*'):ti,ab,kw) OR (('carbon*' NEAR/2 'anhydrase' NEAR/2 'inhibitor*'):ti,ab,kw) OR (('carboxyanhydrase' NEAR/2 'inhibitor*'):ti,ab,kw) OR 'acetazolam*':ti,ab,kw OR 'akzol':ti,ab,kw OR (('ak' NEAR/2 'zol'):ti,ab,kw) OR 'defiltran':ti,ab,kw OR 'diacarb':ti,ab,kw OR 'diamox':ti,ab,kw OR 'diuramide':ti,ab,kw OR 'edemox':ti,ab,kw OR 'glauconox':ti,ab,kw OR 'acetazolamide':ti,ab,kw OR (('huma' NEAR/2 'zolamide'):ti,ab,kw) OR 'benzolamide':ti,ab,kw OR (('cl' NEAR/2 '11366'):ti,ab,kw) OR 'sulfonamide*':ti,ab,kw OR 'methazolamide*':ti,ab,kw OR 'thiadiazole*':ti,ab,kw OR 'daranide':ti,ab,kw OR 'dichlofenamid*':ti,ab,kw OR 'dichlorphenamid*':ti,ab,kw OR 'glauconide':ti,ab,kw OR (('n' NEAR/2 'mrthylacetazolamide'):ti,ab,kw) OR 'carbonic anhydrase inhibitors'/exp OR 'dichlorphenamide'/exp OR 'acetazolamide'/exp OR 'benzolamide'/exp OR 'methazolamide'/exp
**5**	'clinical trial'/exp OR 'clinical trial'/de OR 'randomized controlled trial'/exp OR 'randomized controlled trial'/de OR 'randomization'/exp OR 'randomization'/de OR 'single blind procedure'/exp OR 'single blind procedure'/de OR 'double blind procedure'/exp OR 'double blind procedure'/de OR 'crossover procedure'/exp OR 'crossover procedure'/de OR 'placebo'/exp OR 'placebo'/de OR 'prospective study'/exp OR 'prospective study'/de OR ('randomi?ed controlled' NEXT/1 trial*) OR rct OR 'randomly allocated' OR 'allocated randomly' OR 'random allocation'/exp OR 'random allocation' OR (allocated NEAR/2 random) OR (single NEXT/1 blind*) OR (double NEXT/1 blind*) OR ((treble OR triple) NEAR/1 blind*) OR placebo*
**6**	#3 AND #4 AND #5

Table 3: SCOPUS search strategy

SCOPUS	
#	**Searches**
	(((TITLE-ABS-KEY ((pneumonectomy OR ("pleural disease*") OR empyema* OR hemothrax OR (pleural W/2 (effusion* OR neoplasm *)) OR thoracentesis OR pleurit* OR pneumothorax OR tuberculous))) OR (TITLE-ABS-KEY ((("mountain sickness") OR apnea* OR ("cheyne stokes respiration*") OR breathlessness*) OR ((breath W/2 shortness) OR dyspnea* OR hoarseness* OR hyperventilation* OR ("mouthbreathing*"))))) OR (TITLE-ABS-KEY ((("respiratory depression") OR choking OR ((airway OR nasal) W/2 obstruction*) OR ("hards") OR hypoventilation* OR (sequestration* W/2 pulmonary) OR ("choanal atresia*") OR ("lung malformation adenomatoid*"))))) OR (TITLE-ABS-KEY ((bronchiectasis OR whooping OR tracheitis OR pertuss*) OR ((pleural W/2 disease*) OR (respiration W/2 disorders) OR ("acute chest syndrome") OR apnea OR dyspnea OR ("meconium aspiration syndrome"))))) OR (TITLE-ABS-KEY (("mouth breathing") OR ("respiratory aspiration") OR (respiratory W/2 insufficiency) OR (airway W/2 obstruction) OR (respiratory W/2 paralysis) OR sarcoglycanopathies OR tachypnea OR (" vocal cord dysfunction")))) OR (TITLE-ABS-KEY (("respiratory hypersensitivity") OR ("respiratory system abnormalities") OR ("respiratory tract fistula") OR ("respiratory tract infection*") OR ("respiratory tract neoplasm*") OR ("thoracic disease*"))))) OR ((TITLE-ABS-KEY (("tracheal disease*") OR ("respiratory disease") OR ("bronchial* disease") OR ("pulmonary disease") OR ("lung disease") OR ("silo filller disease") OR ("urbach wiethe disease") OR ("tracheal disease") OR ("mediastina disease") OR ("otolaryngologic* disease") OR ("laryngeal disease") OR ("pharyngeal disease"))))) OR ((TITLE-ABS-KEY (bronchiectas* OR dextrocardia OR sinusitis OR ("ciliary dyskinesia") OR ("pulmonary cancer") OR ("pulmonary neoplasm*") OR (" lung cancer") OR ("lung neoplasm*") OR bronchiolitis* OR bronchitis* OR boop OR pneumonia* OR chondromalacia* OR tracheobronchomegal* OR ("cilia* dyskinesia*") OR ("cilia motility") OR bronchiectasis OR granuloma* OR laryngiti* OR croup OR laryngocele* OR laryngomalacia* OR supraglottiti* OR (subglottic W/2 stenos*) OR ((laryngeal OR larynx) W/2 (perichondriti* OR edema* OR cancer OR neoplasm* OR avulsion* OR transection* OR injur* OR chondromalacia* OR stenos*)))))) OR ((TITLE-ABS-KEY (tuberculos* OR ("vocal cord* dysfunction*") OR ("paradoxical vocal fold") OR laryngismus OR laryngospasm* OR (laryngeal W/2 spasm*) OR aphonia * OR dysphonia* OR ("adenomatoid malformation*") OR ((cystic OR pulmonary OR radiation) W/2 fibrosis)))) OR ((TITLE-ABS-

	KEY (mucoviscidosis OR hemoptys* OR (pulmonary W/2 hypertension*) OR ("acd mpv") OR ("alveolar capillary dysplasia*") OR ("ductus arteriosus") OR ("hht pph1") OR ((lung OR pulmonary) W/2 abscess*) OR blastomycos* OR pneumocyst* OR aspergillosis OR alveoliti* OR ("fancier* lungs") OR ("farmer lungs") OR ("mushroom lungs") OR ("black lungs") OR ("coal lungs") OR ("brown lungs") OR ("trichosporonos disease ") OR ("anti gbm disease") OR ("anti glomerular disease") OR ("hand schuller christian disease") OR ("letterer swie disease") OR ("hashimoto pritzge disease") OR ("beryllium disease") OR ("hamman rich disease") OR ("lung purpra nephritis")))) OR ((TITLE-ABS-KEY (langerhan* OR histocytos* OR reticulendothelios* OR bagassosis OR pneumoconios* OR anthracos* OR anthracosilicos* OR asbestos* OR beryllios* OR byssinos* OR sideros* OR silicos* OR silicotuberculos* OR myelobastin OR bronchitis OR copd OR coad OR (chronic W/2 airflow W/2 obstruction*) OR emphysema* OR (pulmonary W/2 (infection* OR cyst OR hydatid* OR edema* OR pseudotumor* OR collapse OR embolism* OR eosinophili* OR infarction)) OR ("lung injury") OR ("lung carcinoma*") OR (" lung hyperlucent*") OR (" lung collapse") OR ("wet* lung") OR ("meconium aspiration") OR ("bronchopulmonary dysplasia") OR bronchogeni* OR (pulmonary W/2 nodule*) OR (respiratory W/2 aspiration*) OR atelectas*)) OR (TITLE-ABS-KEY (("asthma* syndrome*") OR (" bronchospasm* syndrome*") OR (" aspirin syndrome*") OR (" nsaid syndrome*") OR ("kartagener* syndrome*") OR ("siewert syndrome*") OR ("williams campbell syndrome*") OR ("persistent fetal circulation* syndrome") OR (" hepatopulmonary syndrome*") OR ("caplan syndrome") OR ("goodpasture* syndrome") OR ("pancoast* syndrome") OR ("macleod syndrome") OR ("swyer jame* syndrome") OR (" mendelson syndrome") OR ("aspiration syndrome") OR ("brock* syndrome") OR ("loeffler syndrome") OR ("scimitar syndrome") OR ("pickwickian syndrome"))) OR (TITLE-ABS -KEY (hyalinosis OR (hyaline W/2 disease) OR tachypnea OR anomal* OR ("tapvr ") OR ("total anomalous pulmonary venous return") OR (pulmonary W/2 lesion*))))) AND ((INDEXTERMS ("clinical trials" OR "clinical trials as a topic" OR "randomized controlled trial" OR "Randomized Controlled Trials as Topic" OR "controlled clinical trial" OR "Controlled Clinical Trials" OR "random allocation" OR "Double-Blind Method" OR "Single-Blind Method" OR "Cross-Over Studies" OR "Placebos" OR "multicenter study" OR "double blind procedure" OR "single blind procedure" OR "crossover procedure" OR "clinical trial" OR "controlled study" OR "randomization" OR "placebo")) OR (TITLE-ABS-KEY (("clinical trials" OR "clinical trials as a topic" OR "randomized controlled trial" OR "Randomized Controlled Trials as Topic" OR "controlled clinical trial" OR "Controlled Clinical Trials as Topic" OR "random allocation" OR "randomly allocated" OR "allocated randomly" OR "Double-Blind Method" OR "Single-Blind Method" OR "Cross-Over Studies" OR "Placebos" OR "cross-over trial" OR "single

	blind" OR "double blind" OR "factorial design" OR "factorial trial")))) AND (((TITLE-ABS KEY ((acetazolamide* OR (carbonic* W/2 (dehydratase OR anhydrase) W/2 inhibi tor*) OR (carboxyanhydrase W/2 inhibitor*) OR acetadiazol OR acetazolam* OR a kzol OR defiltran OR diacarb OR diamox OR diuramide))) OR (TITLE-ABS-KEY ((edemox OR glauconox OR glaupax OR (huma W/2 zolamide) OR benzola mide OR (cl "11366") OR sulfonamide* OR thiadiazole* OR daranide OR dichlofe namid* OR dichlorphenamid* OR glauconide OR methazolamide* OR ("n mrthylacetazolamide"))))))

Table 4: Cochrane Central search strategy

**Cochrane Central**	
**#**	**Searches**
**1**	MeSH descriptor: [Alkalosis] explode all trees
**2**	"alkalosis":ti,ab,kw (Word variations have been searched)
**3**	"alkaloses":ti,ab,kw (Word variations have been searched)
**4**	MeSH descriptor: [Hypercapnia] explode all trees
**5**	hypercapni*:ti,ab,kw (Word variations have been searched)
**6**	MeSH descriptor: [Acid-Base Imbalance] explode all trees
**7**	hypoxi*:ti,ab,kw (Word variations have been searched)
**8**	MeSH descriptor: [Hypoxia] explode all trees
**9**	anoxemi* or anoxi* or (oxygen near/2 deficienc*) or hypoxemi*:ti,ab,kw (Word variations have been searched)
**10**	MeSH descriptor: [Ventilators, Mechanical] explode all trees
**11**	ventilator* or respirator* or (artificial W/2 respiration*):ti,ab,kw (Word variations have been searched)
**12**	MeSH descriptor: [Positive-Pressure Respiration] explode all trees
**13**	((positive near/2 pressure near/2 (ventilation* or respiration*)) or (positive near/2 expiratory near/2 pressure)):ti,ab,kw (Word variations have been searched)
**14**	MeSH descriptor: [Intermittent Positive-Pressure Ventilation] explode all trees
**15**	MeSH descriptor: [Noninvasive Ventilation] explode all trees
**16**	NIPPV or BiPAP:ti,ab,kw (Word variations have been searched)

**17**	(respiratory or ventilator) near/2 (failure or insufficiency or depression):ti,ab,kw (Word variations have been searched)
**18**	ippv or (inspiratory near/2 positive near/2 pressure near/2 (ventilation or ventilator)) or aprv or CPAP or ncpap or (positive near/2 airway near/2 pressure):ti,ab,kw (Word variations have been searched)
**19**	MeSH descriptor: [Respiratory Insufficiency] explode all trees
**20**	CO2 near/2 retention:ti,ab,kw (Word variations have been searched)
**21**	(respiratory or bronchial* or pulmonary or lung or (silo near/2 filller) or (urbach near/2 wiethe) or tracheal or mediastinal or pleural or otolaryngologic* or laryngeal or pharyngeal or gilchrist* or larynx) near/2 disease*:ti,ab,kw (Word variations have been searched)
**22**	asthma* or bronchospasm* or ((aspirin or nsaid or kartagener* or siewert or (williams near/2 campbell) or (persistent near/2 fetal near/2 circulation*) or hepatopulmonary or caplan or goodpasture* or pancoast* or macleod or (swyer near/2 jame*) or mendelson or aspiration or brock* or loeffler or (respiratory near/2 distress) or chest or scimitar or pickwickian) near/2 syndrome*) or bronchiectas* or dextrocardia or sinusitis or (ciliary near/2 dyskinesia) or ((pulmonary or lung) near/2 (cancer or neoplasm*)) or bronchiolitis* or bronchitis* or boop or pneumonia* or chondromalacia* or tracheobronchomegal* or (cilia* near/2 (dyskinesia* or motility)) or bronchiectasis or granuloma*:ti,ab,kw (Word variations have been searched)
**23**	((laryngeal or larynx) near/2 (perichondriti* or edema* or cancer or neoplasm* or avulsion*or transection*or injur*or chondromalacia* or stenos*)) or laryngiti* or croup or laryngocele* or laryngomalacia* or supraglottiti* or (subglottic near/2 stenos*):ti,ab,kw (Word variations have been searched)
**24**	tuberculos* or (vocal near/2 cord* near/2 dysfunction*) or (paradoxical near/2 vocal near/2 fold) or laryngismus or laryngospasm* or (laryngeal near/2 spasm*) or aphonia* or dysphonia*or (adenomatoid near/2 malformation*) or ((cystic or pulmonary or radiation) near/2 fibrosis) or mucoviscidosis or hemoptys* or (pulmonary near/2 hypertension*) or (acd near/2 mpv) or (alveolar near/2 capillary near/2 dysplasia*) or (ductus near/2 arteriosus) or (hht near/2 pph1) or ((lung or pulmonary) near/2 abscess*) or blastomycos*.mp. or pneumocyst* or aspergillosis or alveoliti* or ((fancier* or farmer or mushroom or black or coal or brown) near/2 lung*):ti,ab,kw (Word variations have been searched)
**25**	trichosporonos* or (anti near/2 gbm) or (((anti near/2 glomerular) or (hand near/2 schuller near/2 christian) or (letterer near/2 swie) or (hashimoto near/2 pritzge) or beryllium or (hamman near/2 rich)) near/2 disease*) or (lung near/2 purpra near/2 nephritis) or langerhan* or histocytos* or reticulendothelios* or bagassosis or pneumoconios* or anthracos* or anthracosilicos* or asbestos* or beryllios* or byssinos* or sideros* or silicos* or silicotuberculos* or myelobastin or bronchitis or copd or coad or (chronic near/2 airflow near/2 obstruction*) or emphysema* or

	(pulmonary near/2 (infection* or cyst or hydatid* or edema* or granuloma* or pseudotumor* or collapse or embolism* or eosinophili* or infarction)) or (lung near/2 (injur* or carcinoma* or hyperlucent* or collapse or wet*)) or (meconium near/2 aspiration) or (bronchopulmonary near/2 dysplasia) or bronchogeni* or (pulmonary near/2 nodule*) or (respiratory near/2 aspiration*) or atelectas*:ti,ab,kw (Word variations have been searched)
**26**	hyalinosis or (hyaline near/2 membrane near/2 disease*) or tachypnea or anomal* or tapvr or (total near/2 anomalous near/2 pulmonary near/2 venous near/2 return) or (pulmonary near/2 lesion*) or pneumonectomy or (pleural near/2 disease*) or empyema* or hemothrax or (pleural near/2 (effusion* or neoplasm*)) or thoracentesis or pleuri* or pneumothorax or tuberculous or hypoxia* or (mountain near/2 sickness) or apnea* or (cheyne near/2 stokes near/2 respiration*) or breathlessness*:ti,ab,kw (Word variations have been searched)
**27**	(breath near/2 shortness) or dyspnea* or hoarseness* or hyperventilation* or (mouth near/2 breathing*) or (respiratory near/2 depression) or choking or ((airway or nasal) near/2 obstruction*) or hards:ti,ab,kw (Word variations have been searched)
**28**	(hantavirus near/2 associated near/2 respiratory near/2 distress near/2 syndrome*) or hypoventilation* or (sequestration* near/2 pulmonary) or (choanal near/2 atresia*) or (lung near/2 malformation near/2 adenomatoid*) or bronchiectasis or whooping:ti,ab,kw (Word variations have been searched)
**29**	tracheitis or pertuss*:ti,ab,kw (Word variations have been searched)
**30**	MeSH descriptor: [Respiratory Tract Diseases] explode all trees
**31**	MeSH descriptor: [Bronchial Diseases] explode all trees
**32**	MeSH descriptor: [Ciliary Motility Disorders] explode all trees
**33**	MeSH descriptor: [Granuloma, Respiratory Tract] explode all trees
**34**	MeSH descriptor: [Laryngeal Diseases] explode all trees
**35**	MeSH descriptor: [Lung Diseases] explode all trees
**36**	MeSH descriptor: [Nose Diseases] explode all trees
**37**	MeSH descriptor: [Pleural Diseases] explode all trees
**38**	MeSH descriptor: [Acute Chest Syndrome] explode all trees
**39**	MeSH descriptor: [Respiration Disorders] explode all trees
**40**	MeSH descriptor: [Respiratory Distress Syndrome, Newborn] explode all trees
**41**	MeSH descriptor: [Respiratory Distress Syndrome, Adult] explode all trees
**42**	MeSH descriptor: [Hantavirus Pulmonary Syndrome] explode all trees

**43**	MeSH descriptor: [Respiratory Insufficiency] explode all trees
**44**	MeSH descriptor: [Airway Obstruction] explode all trees
**45**	MeSH descriptor: [Granuloma, Laryngeal] explode all trees
**46**	MeSH descriptor: [Hypoventilation] explode all trees
**47**	MeSH descriptor: [Positive-Pressure Respiration] explode all trees
**48**	MeSH descriptor: [Respiratory Paralysis] explode all trees
**49**	MeSH descriptor: [Respiratory System Abnormalities] explode all trees
**50**	MeSH descriptor: [Respiratory Tract Fistula] explode all trees
**51**	MeSH descriptor: [Respiratory Tract Infections] explode all trees
**52**	MeSH descriptor: [Respiratory Tract Neoplasms] explode all trees
**53**	MeSH descriptor: [Thoracic Diseases] explode all trees
**54**	MeSH descriptor: [Tracheal Diseases] explode all trees
**55**	acetazolamide* or (carbon* near/2 (dehydratase or anhydrase) near/2 inhibitor*) or (carboxyanhydrase near/2 inhibitor*) or acetadiazol or acetazolam* or akzol or (ak near/2 zol) or defiltran or diacarb or diamox or diuramide or edemox or glauconox or glaupax or (huma near/2 zolamide) or benzolamide or (cl near/2 "11366") or sulfonamide* or thiadiazole* or daranide or dichlofenamid* or dichlorphenamid* or glauconide or methazolamide* or (n near/2 mrthylacetazolamide):ti,ab,kw (Word variations have been searched)
**56**	MeSH descriptor: [Carbonic Anhydrase Inhibitors] explode all trees
**57**	MeSH descriptor: [Acetazolamide] explode all trees
**58**	MeSH descriptor: [Benzolamide] explode all trees
**59**	MeSH descriptor: [Dichlorphenamide] explode all trees
**60**	MeSH descriptor: [Methazolamide] explode all trees
**61**	#1 or #2 or #3 or #4 or #5 or #6 or #7 or #8 or #9 or #10 or #11 or #12 or #13 or #14 or #15 or #16 or #17 or #18 or #19 or #20 or #21 or #22 or #23 or #24 or #25 or #26 or #27 or #28 or #29 or #30 or #31 or #32 or #33 or #34 or #35 or #36 or #37 or #38 or #39 or #40 or #41 or #42 or #43 or #44 or #45 or #46 or #47 or #48 or #49 or #50 or #51 or #52 or #53 or #54
**62**	#55 or #56 or #57 or #58 or #59 or #60
**63**	#61 and #62

Table 5: Funding and conflict of interest of authors in included trials:

Study name:	Funding and Conflict of interest of authors:
Faisy 2016 (6)	- French ministry of health, Sanofi/Aventis, and the French National Committee for Health Research<br><br>- Conflict of Interest Disclosure: all authors have completed and submitted the ICMJE Form for Disclosure of Potential Conflicts of Interest.<br>Dr.Richard reported receiving travel expenses from Fisher and Paykel.<br>Dr.Sanchez reported receiving grants from Bayer, Daiichi Sankyo, Boehringer Ingelheim, and Actellion; personal fees from Bayer, Daiichi Sankyo, Bristol-Myers Squibb, Boehringer Ingelheim, and Actellion; and nonfinancial support from Bayer, Chiesi, GlaxoSmithKline, and Actelion.<br>No other disclosures were reported
Rialp Cervera 2017 (8)	- Work supported by Conselleria de Salut i Consum del Govern de les Illes Balears [grant number DGAVAL_PI_019 HSLL] and by Conselleria d'Innovacio, Interior i Justícia del Govern de les Illes Balears [grant number AAEE0160/09].<br>- None of the authors have any conflict of interest with the study
Nelson, 1965 (12)	- Wellcome foundation, Northern Ireland Hospitals Authorities, Royal Victoria Hospital endowment fund, Medical Research Council<br>- Conflict of interest was not mentioned
Hacki,1983 (15)	- Not specified
Vos, 1994 (29)	- Supported by a grand from the Dutch asthma foundation<br><br>- Conflict of interest was not mentioned
Gulsvik, 2013 (28)	- Reported as non-funded<br><br>- None of the authors have any support from any organization for the submitted work, no financial relationships with any organizations that might have an interest in the submitted work or any other relationships or activities that could appear to have influenced thee submitted work.

Figure 1: Risk of bias summary. (+): low risk of bias, (-): high risk of bias, (?): unclear risk of bias

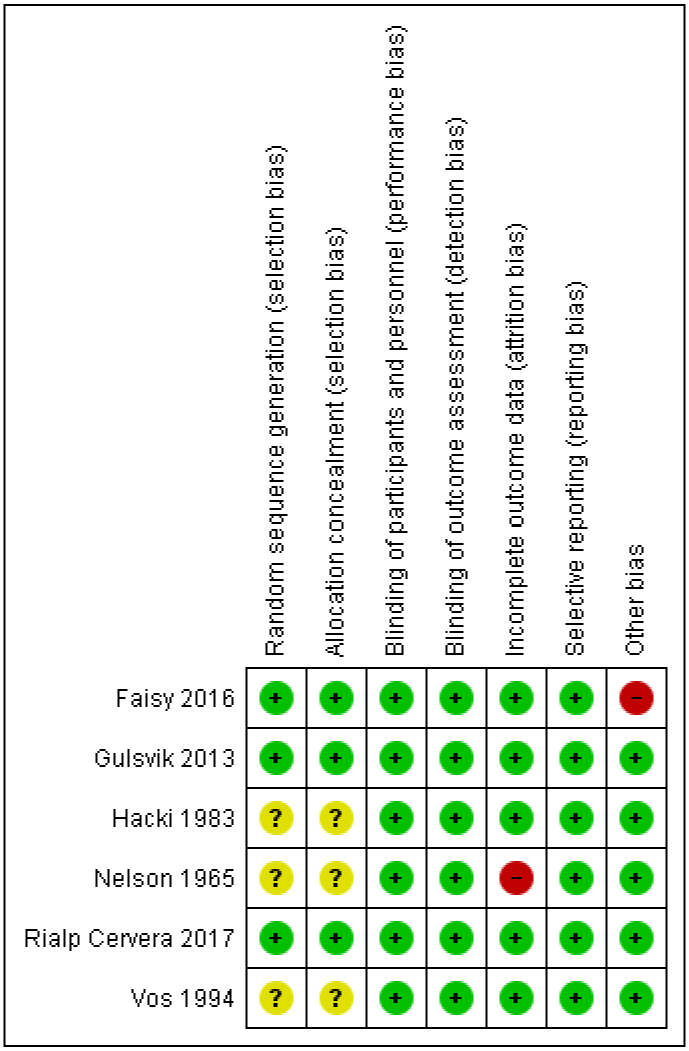

Figure 2: Forest plot for the effect of CAI vs control on mortality

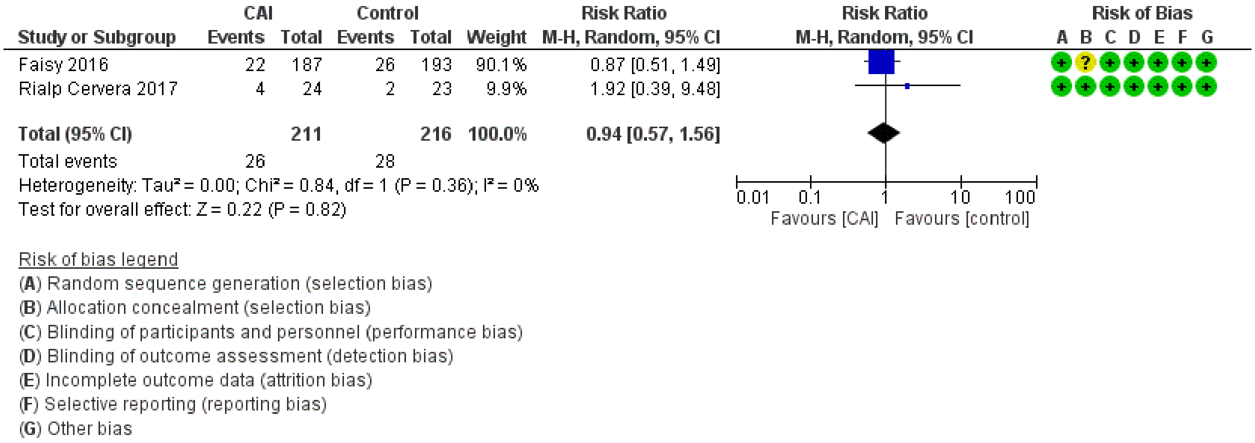

Study or Subgroup	CAI Events	CAI Total	Control Events	Control Total	Weight	Risk Ratio M-H, Random, 95% CI	Risk of Bias A B C D E F G
Faisy 2016	22	187	26	193	90.1%	0.87 [0.51, 1.49]	+ ? + + + + +
Rialp Cervera 2017	4	24	2	23	9.9%	1.92 [0.39, 9.48]	+ + + + + + +
**Total (95% CI)**		**211**		**216**	**100.0%**	**0.94 [0.57, 1.56]**	
Total events	26		28				

Heterogeneity: Tau² = 0.00; Chi² = 0.84, df = 1 (P = 0.36); I² = 0%
Test for overall effect: Z = 0.22 (P = 0.82)

Risk of bias legend
(A) Random sequence generation (selection bias)
(B) Allocation concealment (selection bias)
(C) Blinding of participants and personnel (performance bias)
(D) Blinding of outcome assessment (detection bias)
(E) Incomplete outcome data (attrition bias)
(F) Selective reporting (reporting bias)
(G) Other bias

Figure 3: Forest plot for the effect of CAI vs control on duration of hospital stay

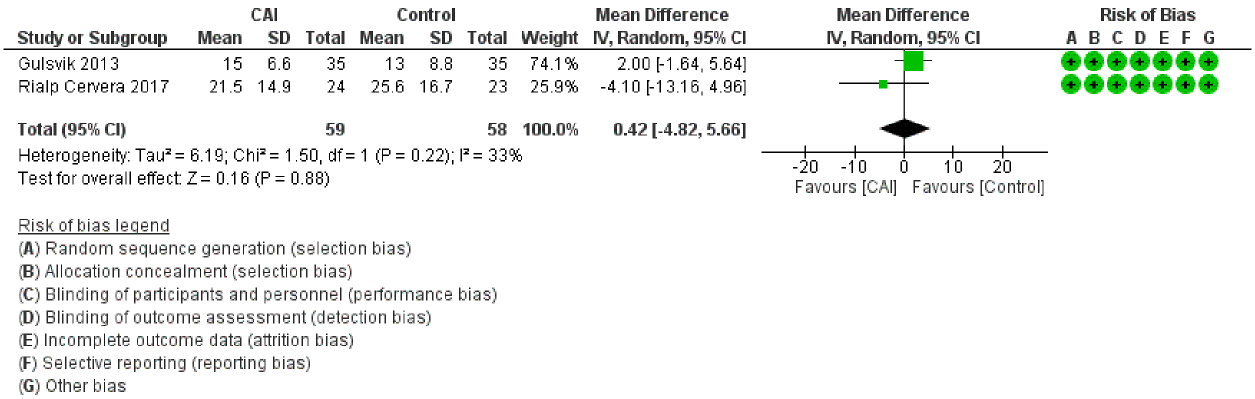

Study or Subgroup	CAI Mean	CAI SD	CAI Total	Control Mean	Control SD	Control Total	Weight	Mean Difference IV, Random, 95% CI	Risk of Bias A B C D E F G
Gulsvik 2013	15	6.6	35	13	8.8	35	74.1%	2.00 [-1.64, 5.64]	+ + + + + + +
Rialp Cervera 2017	21.5	14.9	24	25.6	16.7	23	25.9%	-4.10 [-13.16, 4.96]	+ + + + + + +
**Total (95% CI)**			**59**			**58**	**100.0%**	**0.42 [-4.82, 5.66]**	

Heterogeneity: Tau² = 6.19; Chi² = 1.50, df = 1 (P = 0.22); I² = 33%
Test for overall effect: Z = 0.16 (P = 0.88)

Risk of bias legend
(A) Random sequence generation (selection bias)
(B) Allocation concealment (selection bias)
(C) Blinding of participants and personnel (performance bias)
(D) Blinding of outcome assessment (detection bias)
(E) Incomplete outcome data (attrition bias)
(F) Selective reporting (reporting bias)
(G) Other bias

Figure 4: Forest plot for the effect of CAI vs control on duration of mechanical ventilation

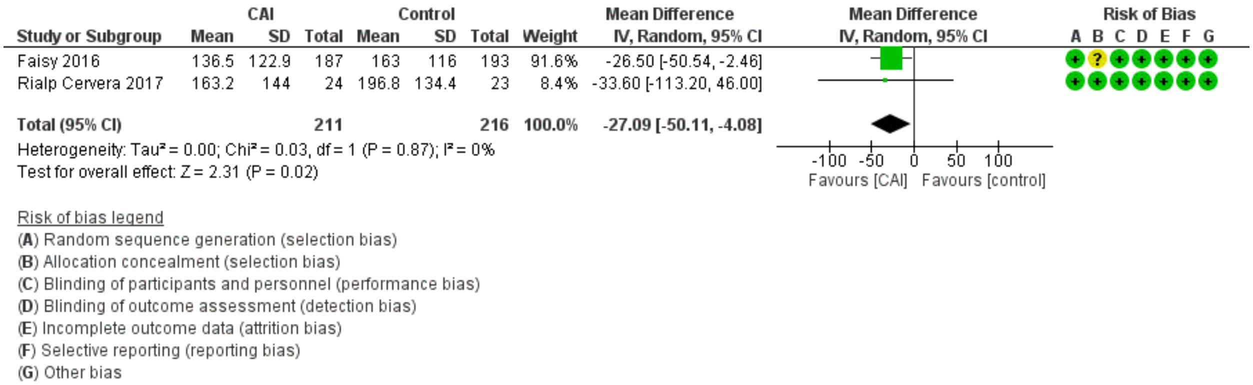

Study or Subgroup	CAI Mean	CAI SD	CAI Total	Control Mean	Control SD	Control Total	Weight	Mean Difference IV, Random, 95% CI	Risk of Bias A B C D E F G
Faisy 2016	136.5	122.9	187	163	116	193	91.6%	-26.50 [-50.54, -2.46]	+ ? + + + + +
Rialp Cervera 2017	163.2	144	24	196.8	134.4	23	8.4%	-33.60 [-113.20, 46.00]	+ + + + + + +
**Total (95% CI)**			**211**			**216**	**100.0%**	**-27.09 [-50.11, -4.08]**	

Heterogeneity: Tau² = 0.00; Chi² = 0.03, df = 1 (P = 0.87); I² = 0%
Test for overall effect: Z = 2.31 (P = 0.02)

Risk of bias legend
(A) Random sequence generation (selection bias)
(B) Allocation concealment (selection bias)
(C) Blinding of participants and personnel (performance bias)
(D) Blinding of outcome assessment (detection bias)
(E) Incomplete outcome data (attrition bias)
(F) Selective reporting (reporting bias)
(G) Other bias

Figure 5: Forest plot for the effect of CAI vs control on PaCO2 using standardized mean difference.

Study or Subgroup	CAI Mean	CAI SD	CAI Total	Control Mean	Control SD	Control Total	Weight	Std. Mean Difference IV, Random, 95% CI
Faisy 2016	-0.5	2	187	-0.2	1.77	193	21.0%	-0.16 [-0.36, 0.04]
Gulsvik 2013	-2.2	2.5	35	-3.3	1.8	35	19.5%	0.50 [0.02, 0.98]
Hacki 1983	-10.1	3	5	-1.7	1.9	8	8.6%	-3.31 [-5.20, -1.42]
Nelson 1965	55.1	6.8	10	60.5	7.5	10	15.9%	-0.72 [-1.63, 0.19]
Rialp Cervera 2017	48	3.7	24	55	9.7	23	18.5%	-0.95 [-1.55, -0.34]
Vos 1994	-3.7	3.3	17	2.2	2.2	18	16.5%	-2.07 [-2.91, -1.23]
**Total (95% CI)**			**278**			**287**	**100.0%**	**-0.85 [-1.57, -0.13]**

Heterogeneity: Tau² = 0.63; Chi² = 44.49, df = 5 (P < 0.00001); I² = 89%
Test for overall effect: Z = 2.32 (P = 0.02)

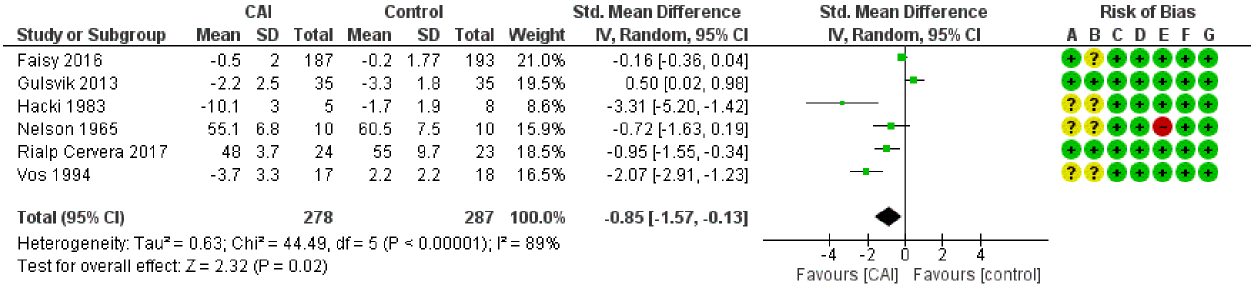

Risk of bias legend
(A) Random sequence generation (selection bias)
(B) Allocation concealment (selection bias)
(C) Blinding of participants and personnel (performance bias)
(D) Blinding of outcome assessment (detection bias)
(E) Incomplete outcome data (attrition bias)
(F) Selective reporting (reporting bias)
(G) Other bias

Figure 6: Forest plot for the effect of CAI vs control on PaO2 using standardized mean difference.

Study or Subgroup	CAI Mean	CAI SD	CAI Total	Control Mean	Control SD	Control Total	Weight	Std. Mean Difference IV, Random, 95% CI
Faisy 2016	7.8	16.2	187	3.5	14.1	193	23.6%	0.28 [0.08, 0.49]
Gulsvik 2013	10.5	2	35	6	2	35	22.6%	2.23 [1.62, 2.83]
Hacki 1983	15.4	2.6	5	-3.5	3.6	8	11.5%	5.37 [2.66, 8.09]
Rialp Cervera 2017	223	84.9	24	207	79.5	23	22.7%	0.19 [-0.38, 0.76]
Vos 1994	14.2	3.2	17	3	2.1	18	19.6%	4.07 [2.86, 5.28]
**Total (95% CI)**			**268**			**277**	**100.0%**	**2.03 [0.74, 3.32]**

Heterogeneity: Tau² = 1.81; Chi² = 82.50, df = 4 (P < 0.00001); I² = 95%
Test for overall effect: Z = 3.09 (P = 0.002)

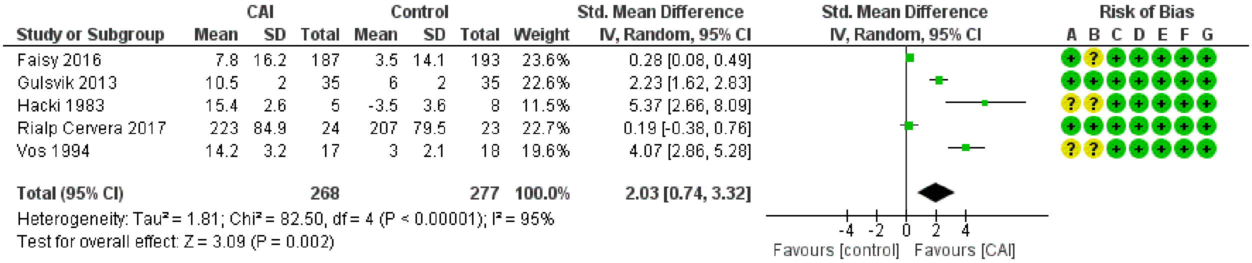

Risk of bias legend
(A) Random sequence generation (selection bias)
(B) Allocation concealment (selection bias)
(C) Blinding of participants and personnel (performance bias)
(D) Blinding of outcome assessment (detection bias)
(E) Incomplete outcome data (attrition bias)
(F) Selective reporting (reporting bias)
(G) Other bias

Figure 7: Forest plot for the effect of CAI vs control on serum bicarbonate using standardized mean difference.

Study or Subgroup	CAI Mean	CAI SD	CAI Total	Control Mean	Control SD	Control Total	Weight	Std. Mean Difference IV, Random, 95% CI	Risk of Bias A	B	C	D	E	F	G
Faisy 2016	-3	1	187	0.3	1.1	193	19.1%	-3.13 [-3.43, -2.83]	+	?	+	+	+	+	+
Gulsvik 2013	-8.62	0.81	35	-2.15	0.77	35	16.3%	-8.10 [-9.55, -6.64]	+	+	+	+	+	+	+
Hacki 1983	-6.8	0.79	5	-3.4	0.55	8	12.4%	-4.88 [-7.39, -2.37]	?	?	+	+	+	+	+
Nelson 1965	26.5	2.5	10	31.6	3.6	10	17.6%	-1.58 [-2.61, -0.54]	?	?	+	+	−	+	+
Rialp Cervera 2017	29	2.5	24	34	3.6	23	18.5%	-1.59 [-2.26, -0.93]	+	+	+	+	+	+	+
Vos 1994	-6.7	1.61	17	0.3	0.74	18	16.0%	-5.51 [-7.04, -3.99]	?	?	+	+	+	+	+
**Total (95% CI)**			**278**			**287**	**100.0%**	**-3.98 [-5.47, -2.49]**							

Heterogeneity: Tau² = 3.00; Chi² = 84.78, df = 5 (P < 0.00001); I² = 94%
Test for overall effect: Z = 5.24 (P < 0.00001)

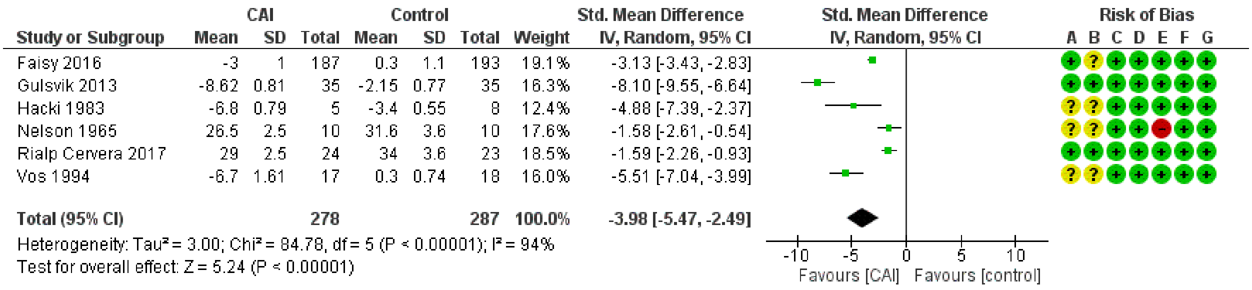

Risk of bias legend
(A) Random sequence generation (selection bias)
(B) Allocation concealment (selection bias)
(C) Blinding of participants and personnel (performance bias)
(D) Blinding of outcome assessment (detection bias)
(E) Incomplete outcome data (attrition bias)
(F) Selective reporting (reporting bias)
(G) Other bias

Figure 8: Forest plot for the effect of CAI vs control on pH using standardized mean difference.

Study or Subgroup	CAI Mean	CAI SD	CAI Total	Control Mean	Control SD	Control Total	Weight	Std. Mean Difference IV, Random, 95% CI	Risk of Bias A	B	C	D	E	F	G
Faisy 2016	0	0.02	187	0.01	0.01	193	21.4%	-0.63 [-0.84, -0.43]	+	?	+	+	+	+	+
Gulsvik 2013	-0.09	0.01	35	-0.003	0.01	35	18.9%	-8.60 [-10.14, -7.06]	+	+	+	+	+	+	+
Hacki 1983	-0.05	0.02	5	-0.04	0.02	8	20.0%	-0.47 [-1.60, 0.67]	?	?	+	+	+	+	+
Rialp Cervera 2017	7.4	0.02	24	7.41	0.03	23	21.1%	-0.39 [-0.97, 0.19]	+	+	+	+	+	+	+
Vos 1994	-0.07	0.01	17	-0.01	0.01	18	18.7%	-5.86 [-7.46, -4.26]	?	?	+	+	+	+	+
**Total (95% CI)**			**268**			**277**	**100.0%**	**-3.03 [-4.94, -1.11]**							

Heterogeneity: Tau² = 4.45; Chi² = 141.79, df = 4 (P < 0.00001); I² = 97%
Test for overall effect: Z = 3.10 (P = 0.002)

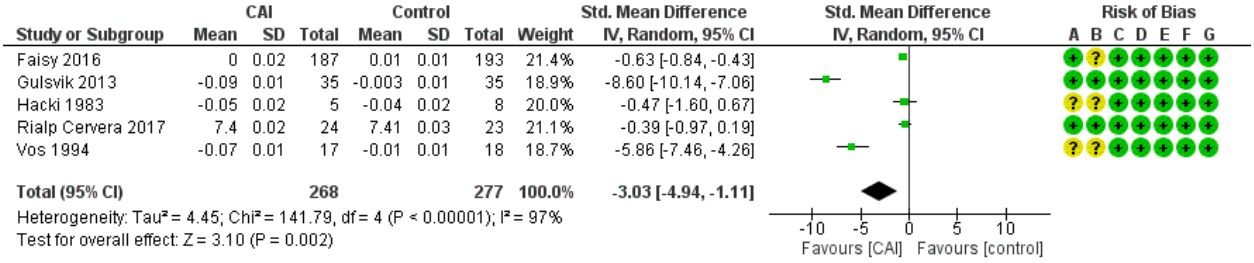

Risk of bias legend
(A) Random sequence generation (selection bias)
(B) Allocation concealment (selection bias)
(C) Blinding of participants and personnel (performance bias)
(D) Blinding of outcome assessment (detection bias)
(E) Incomplete outcome data (attrition bias)
(F) Selective reporting (reporting bias)
(G) Other bias
